# Digital treatment for insomnia in adolescents: study protocol for a randomized controlled trial comparing digital cognitive behavioral therapy for insomnia to sleep hygiene

**DOI:** 10.3389/frcha.2026.1686491

**Published:** 2026-05-01

**Authors:** Beke Ralfs, Sarah Fee Meschkat, Telke Schoone, Hannah Brauer, Jan-Henrik Rieck, Manuel Munz, Charlotte Josephine Müller, Leonie Franziska Maurer, Alexander Prehn-Kristensen

**Affiliations:** 1 Institut for Child and Adolescent Psychiatry, Centre for Integrative Psychiatry, School of Medicine, Christian-Albrechts University Kiel, Kiel, Germany; 2 Clinic for Child and Adolescent Psychiatry, Psychotherapy and Psychosomatics, Centre for Integrative Psychiatry, School of Medicine, Kiel, Germany; 3 Science, Mementor DE GmbH, Leipzig, Germany; 4 Department of Psychology, Faculty of Human Sciences, MSH Medical School Hamburg—University of Applied Sciences and Medical University, Hamburg, Germany

**Keywords:** adolescents, avatar, cognitive behavioral therapy, digital, insomnia, internet, randomized controlled trial, virtual coach

## Abstract

**Background:**

Insomnia is highly prevalent among adolescents. It is associated with various mental and physical disorders and impairments in daytime functioning. Cognitive behavioral therapy for insomnia (CBT-I) is well supported by evidence and the guideline recommended treatment. Yet only few adolescents receive access due to limited health care resources. Digital delivery of CBT-I may tackle this care gap and has shown promising results in the adult population. However, evidence in the adolescent population is still preliminary. The aim of this randomized controlled trial is to establish whether digitally delivered CBT-I for insomnia is effective in adolescents, when compared to sleep hygiene advice.

**Methods:**

This trial is a monocentric, national, prospective randomized controlled trial aiming to recruit 66 adolescent participants (14–17 years) meeting criteria for insomnia disorder in Germany. Participants will be randomized (1:1) to either 12 weeks of digital CBT-I (*somnio junior)* or sleep hygiene advice. Outcomes will be collected at baseline, 6- and 12-weeks post-randomization. The primary endpoint is self-reported insomnia severity using the Insomnia Severity Index at 12 weeks. Secondary endpoints include symptoms of depression, anxiety, daytime sleepiness and health-related quality of life. Analyzes will be intention-to-treat.

**Discussion:**

The results of this study could make the case for the introduction of digital CBT-I for adolescents in primary care in Germany, increasing access to evidence-based treatment for adolescents with insomnia disorder. Treating insomnia might also positively affect associated mental disorders and physical health problems.

**Clinical Trial Registration:**

https://www.bfarm.de/DE/Das-BfArM/Aufgaben/Deutsches-Register-Klinischer-Studien/_node.html, identifier DRKS00033527.

## 1 Background

Insomnia, i.e., difficulty falling asleep and staying asleep, is widespread among adolescents. Depending on the underlying diagnostic criteria, prevalence ranges from 4% to 39% (1). A significantly higher female prevalence emerges after puberty: 23.6% of female and 12.5% of male adolescents fulfill the DSM-5 criteria for insomnia disorder (2, 3). Symptoms of insomnia disorder are described in the DSM-V as “a predominant complaint of dissatisfaction with sleep quantity or quality […] that causes clinically significant distress or impairment in social, occupational, educational, academic, behavioral, or other important areas of functioning” (4). Insomnia can occur at any point in life, but adolescents are particularly vulnerable: due to a puberty-related change in chronotype based on changes in melatonin secretion but also on participation in social events, media use, or other forms of sleep incompatible behavior in the evening/nights making insomnia symptoms in adolescents more likely (5, 6). Inherent to the diagnosis, insomnia is associated with several problems, such as complications with family, social and school or work environment (7–11). Further, it is a risk factor for several physical health concerns and increased all-cause mortality (1, 12, 13). Dysregulated sleeping behavior is generally associated with social-emotional and behavioral problems (14, 15). More specifically, people suffering from insomnia are more vulnerable to developing mental disorders such as depression or anxiety disorder (16).

Insomnia and associated emotional and behavioral problems bidirectionally influence each other and have many mutual underlying factors (12, 17–19). This highlights the importance of adequately treating insomnia as this might also positively influence associated problems (1).

The current guidelines for the treatment of insomnia in children and adolescents recommend behavioral therapy as the method of first choice (20). Cognitive-behavioral therapy for insomnia (CBT-I) in adolescents consists of psychoeducation, mindfulness exercises, sleep hygiene, bedtime restriction, sleep diary and stimulus control (21). Treatment with CBT-I is well-supported by empirical evidence. Meta-analyses assessing CBT-I show robust and long-lasting effects in reducing symptoms of insomnia, daytime functioning and sleep-related outcomes such as sleep onset latency, total sleep time, and sleep efficiency in adults (22) as well as in adolescents (22, 23). Blake and colleagues (24) were able to trace this back to a reduced pre-sleep hyperarousal. Similar results can be found for children (25) and for different cultures and ethnicities (26, 27). Despite the promising prospects of CBT-I, data from health insurances (28) and results from a survey among health care professionals (29) indicate that only a minority of adult patients with insomnia receive access. This might be due to the lack of trained specialists, a lack of knowledge about the possibility of psychotherapeutic treatment of insomnia, and the time expenditure of psychotherapeutic treatment. Although comparable data on CBT-I uptake in child and adolescent populations are lacking, the broader mental-health literature shows that many adolescents who would benefit from psychological treatment do not receive timely and appropriate care. Studies identify a range of attitudinal and structural barriers, including stigma, embarrassment, confidentiality concerns, limited mental-health literacy, and a preference for self-reliance (30).

For adults, there is substantial evidence supporting the effectiveness of digital CBT-I (dCBT-I) (31–33). The use of dCBT-I therefore offers a cost-efficient (34), low-threshold and accessible solution to the treatment gap (35–38). For adolescents, a recent meta-analysis on eight RCTs of CBT-I in adolescents found large treatment effects for insomnia severity [standardized mean difference (SMD) = −1.06], when compared to control (23). Yet, digitally delivered CBT-I interventions for adolescents are barely investigated and, to date, considered preliminary with only four RCTs reporting beneficial effect on sleep and insomnia, when compared to control (39, 40). As discussed by Werner-Seidler and colleagues (41), adolescents may even perceive digital therapy options as more appealing, as they are generally more familiar and comfortable with using digital technologies. Moreover, barriers that often discourage adolescents from seeking help, such as stigma, may be less prominent in digitally delivered interventions. In summary, there is a significant gap in the need and availability of guideline recommended treatment for insomnia in adolescents. Digital solutions may have large potential but are yet lacking substantial evidence. To address this need, a digital application, namely *somnio junior* (mementor DE GmbH), was specifically developed to be investigated, and if proven to be effective, to be integrated into the German health care system.

The primary aim of this randomized controlled trial is to elucidate whether dCBT-I (intervention group, IG) is effective in adolescents diagnosed with insomnia, when compared to sleep hygiene advice (control group, CG).

It is hypothesized that the self-reported insomnia severity (primary endpoint) will decrease in participants of the IG compared to the CG at 12-weeks post-randomization. As secondary endpoints, a decline in symptoms of insomnia at 6-weeks post-randomization and, at 12-weeks post-randomization, a decline of depression, anxiety, daytime sleepiness and an increase in health-related quality of life is expected in the IG, when compared to CG. All variables are measured using self-report questionnaires.

## 2 Methods

### 2.1 Study design and setting

**Figure 1:**
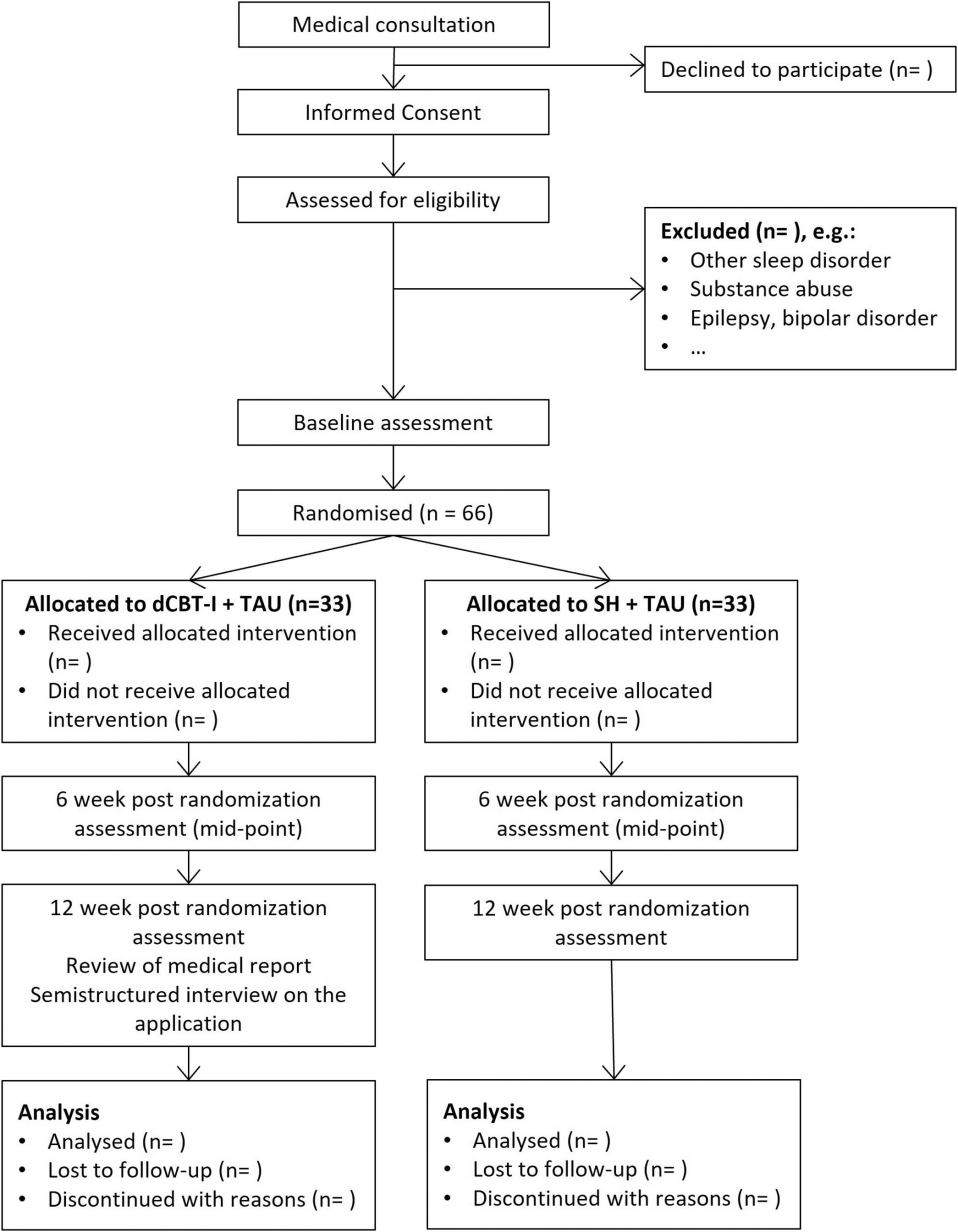
Trial flow.

The current study is a monocentric, national, prospective randomized controlled trial evaluating the effectiveness of the newly developed digital application *somnio junior* in reducing symptoms of insomnia in adolescents. During the study, all participants will be permitted to continue their usual medical and therapeutic care (treatment as usual; TAU).A two-arm study design with *N* = 66 participants is used with an IG (dCBT-I + TAU) and CG (sleep hygiene + TAU) with pre-midpoint-post assessment. At baseline (t0) and post assessment (t2), data on all primary (insomnia severity) and secondary (depression, anxiety, daytime sleepiness, health-related quality of life) parameters are collected through self-assessment questionnaires. At midpoint assessment (t1) only the insomnia severity is assessed. In t1 and t2 participants are asked for any adverse events. For the 12-week study period, the IG has full access to the digital application *somnio junior*, while participants of the CG receive a flyer with advice on sleep hygiene (SH) (“*Your top 10 rules for better sleep*”, Additional File S4). The study process is illustrated in Figure 1. Participants of the CG receive access to the digital application after completion of all study procedures**.**

The study center is the Institute of Child and Adolescent Psychiatry at the Center for Integrative Psychiatry, School of Medicine in Kiel. The clinical trial is conducted entirely online. All visits and interviews are held via the video conferencing platform BigBlueButton (BBB) hosted by the Universitýs Computing Center and questionnaires are filled out online directly via the Electronic-Data-Capture-System (EDC). For instant technical support if required, participants are asked to fill out the questionnaires in the EDC, while staying in the online conference room. To ensure privacy, both, participants and investigators will turn off their camera and are muted.

The study protocol was registered in the German Clinical Trials Register (Deutsche Register Klinischer Studien; DRKS) (DRKS00033527; registration date 25.07.2024), approved by the Ethics Committee (Faculty of Medicine at the University of Kiel, 16.07.2024) and authorized by the German Federal Institute for Drugs and Medical Devices (BfArM, 04.10.2024). A SPIRIT-Checklist (Additional File S1) is provided (42).

A central gender-stratified block randomization with variable block size (2–4) is selected as the randomization procedure. This way, participants are equally distributed between IG and CG regarding gender, being the only stratification variable. Randomization will be implemented through the EDC by external staff of the Center of Clinical Trials (Zentrum für Klinische Studien; ZKS) in Kiel and the allocation sequence is concealed from the research team until interventions are assigned. After completion of the baseline assessment, allocation details will be communicated to participants by members of the research team. As this is an open-label study, both participants and research team members will be aware of the group assignments. The participant information sheet will explain that the study is comparing two sleep intervention programs but will not disclose the study hypothesis. Participants will complete outcomes remotely and without the presence of an investigator, although research staff monitor outcome ratings to assist in case of technical problems. Although blinding the research team is not feasible, the trial statisticians will remain blinded to group assignments.

### 2.2 Treatment as usual (TAU)

German guidelines for persistent insomnia in adolescents recommend psychoeducation, sleep hygiene, relaxation techniques, stimulus control, cognitive strategies, and bedtime restriction (20). Despite the high prevalence of insomnia in adolescents, it remains unclear, how many adolescents diagnosed with insomnia receive guideline-based treatment. In the present study, TAU is defined as any ongoing medical or psychological care participants receive outside the study (existing psychotherapy, medication). TAU is recorded at baseline, midpoint, and post-intervention using a structured form documenting treatment type, frequency, and any changes during the study. TAU will be summarized descriptively but not included as a covariate, as randomization is expected to balance TAU across groups.

### 2.3 Digital application *somnio junior*

**Figure 2:**
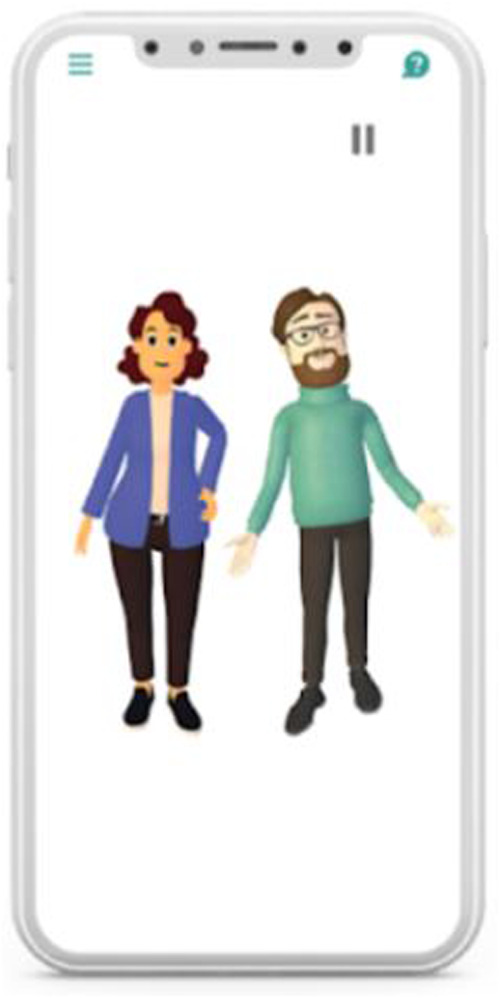
Digital sleep avatar Olivia and Albert.

The software application *somnio junior*, a product of the company mementor DE GmbH, implements contents of CBT-I for the treatment of insomnia in adolescents aged 14 to 17. A similar program (*somnio*) is already used for treating adults with insomnia in Germany (through prescription by practitioners), which showed large effect sizes in reducing insomnia severity compared to CG which were stable over a 12 months-period (43, 44). Contents of somnio were adjusted to age-appropriate requirements, reviewed by experts in the field, and tested by adolescents for usability and feasibility, whose feedback was included in the final stage of development. *somnio junior* comprises 14 educational modules, each taking 5–30 min, is fully automated and delivered by an interactive and animated avatar, who can be chosen (“Albert” or “Olivia”; Figure 2). The application can be accessed through a web browser or mobile application. New content is unlocked successively upon completion of previous modules and sleep diary entries. In addition, subsequent modules offer relapse prevention and help to consolidate previously learned content. Regular, personalised goal modules are implemented to review achievements and progress in the programme.

Table 1 shows an overview and description of the dCBT-I modules implemented in *somnio junior*.

**Table 1:** Overview and description of dCBT-I modules during the intervention.

Endpoints	Module	Description
1.	Introduction	In the introductory module, users meet their personal sleep expert Albert or Olivia for the first time. They ask for details about their current sleep and the type and extent of their sleep complaints.
2.	Sleep journal	The digital sleep diary consists of the morning and evening log. It collects important data on sleep behavior, which is later used to analyze sleep development (bedtime, sleep time and sleep efficiency). This module explains the purpose and use of the diary.
3.	Sleep knowledge	Important information on the topic of sleep is conveyed in an age-appropriate way. This includes scientifically sound findings on the phases of sleep, the sleep rhythm, the optimum amount of sleep and other essential basics. Sleep myths are debunked and fears and worries that prevent sleep are reduced.
4.	Practical exercise 1	Knowledge learned from the previous modules is tested using everyday examples with young characters. The users slip into the role of the sleep expert and advise the characters on their sleep problems.
Goal module		
5.	Cycle of insomnia	How does a sleep disorder develop? What factors contribute to your sleep problems persisting? These questions are answered with the help of the cycle of insomnia.
6.	Sleeping hours	This module teaches the relevance of a regular sleep-wake cycle. Bedtime reduction is introduced as an effective intervention for the treatment of sleep onset and sleep-through disorders. A sleep window is suggested on the basis of previous sleep diary data and the results of the sleep type test. If contraindicated, the bedtime restriction is omitted.
Goal module		
7.	Sleep behavior 1	This module clarifies which factors can have a positive or negative effect on sleep (“sleep hygiene rules”). Misconceptions are corrected and the sleep expert provides support in integrating sleep-promoting behaviors into everyday life. The principle of stimulus control is introduced.
8.	Relaxation	Progressive muscle relaxation (PME) is introduced as a relaxation technique. It is one of the most effective relaxation techniques, easy to learn and easy to integrate into everyday life.
Goal module		
9.	Sleep behavior 2	Factors that influence sleep such as daily activities, diet and substances consumed are discussed. Risks are pointed out and alternative, sleep-promoting behaviors are shown. Users are advised to establish a sleep routine.
10.	Mindfulness	What is mindfulness, what does it do and how can it be practiced? This module introduces mindfulness-based stress reduction.
Goal module		
11.	Practical exercise 2	Knowledge learned from the previous modules is tested using everyday examples with young characters. The users slip into the role of the sleep expert and advise the characters on their sleep problems.
12.	Thoughts	The aim of the module is to identify thoughts that hinder sleep and to practice dealing with them in a way that promotes sleep. This “cognitive restructuring” aims to identify and change unrealistic expectations regarding sleep and misconceptions about the sleep disorder.
Goal module		
13.	Everyday decisions	This module focuses on typical everyday situations in which those affected often exhibit so-called safety behavior. Key aspects include managing rumination, seeking social support, and addressing daytime habits such as napping, maintaining supportive daily routines, and engaging in daytime activities. In addition, the topics sleep pressure and nighttime clock-watching are addressed.
14.	Mindfulness	The knowledge of mindfulness is further consolidated and the sleep expert addresses the user's difficulties individually.
15.	Review	Users are given a comprehensive review of sleep training and can put their sleep knowledge to the test in a quiz format.
16.	Accompaniment	After completing the relevant KVT-I modules, the progression of the most important sleep parameters and insomnia symptoms is re-evaluated at regular intervals. Mood and motivation are assessed and support is offered in the event of difficulties.

A medical report is automatically generated and can be downloaded by the participants as a PDF-document. The medical report includes information on the development of different sleep parameters during the course of the intervention (e.g., sleep efficiency). This analysis can be shared by the user with physicians or therapists so that they are able to evaluate the effectiveness of the intervention together with the adolescent patient.

### 2.4 Participants

We aim to recruit participants aged 14–17 years nationwide through flyers (containing study contact details) in regular care practices, clinics and through online advertisement in social media. Flyers and online advertisement link to the study homepage, which contains all information about the study. Since insomnia is more prevalent in female than in male adolescents [f:m = 3:1 (45)], we expect a similar gender distribution in our study sample.

#### 2.4.1 Eligibility criteria

Eligibility criteria comprise an age of 14 to 17 years, the diagnosis of insomnia according to DSM-5 criteria, sufficient knowledge of the German language, access to a smartphone or tablet with internet connection, and ability to fully participate, in terms of both scope and content, in the study in the given time frame (i.e., able to adhere to study procedures). Patients with self-reported (by themselves or by their parents) epilepsy, bipolar disorder, addiction (including media-related disorders), acute psychiatric hospitalization in the past four weeks or acute suicidal behavior are excluded from participation. Further, sleep problems due to physiological disorders such as patients suffering from chronic pain are excluded, since the proportion of behavioral factors on their sleep problems cannot be determined reliably. The same applies to hypersomnia, sleep-wake rhythm disorder, sleepwalking, night terrors, nightmares, obstructive sleep apnea, restless legs syndrome or narcolepsy. Other comorbidities, such as depression or anxiety disorders, are recorded but do not affect eligibility. Lastly, participants may not take part in another clinical trial at the same time. Comorbidities, medications, and therapy are recorded and will be summarized descriptively, but they will not be included as covariates, as randomization is expected to balance these factors across groups. To ensure participant safety, this information is collected by a physician, and participants are instructed to seek appropriate support and to contact the study team in the event of pregnancy or the emergence of suicidal thoughts.

### 2.5 Procedure

Persons interested in the study can contact the study team via the study website by e-mail or telephone to obtain further information about the study. The study team will respond to the inquiries and will arrange an initial, direct informational interview with the legal guardians and the adolescent via video conference if they are still interested in participating in the study. Here, potential participants and their custodians are informed about the study procedure by the trial physician. Afterwards they may give their informed consent (IC, see Additional Files S2, S3) via their qualified signature on the online platform “DocuSign”. This program will check the identity of the person signing the informed consent form by validating their passport or ID. Participants are enrolled year-round, and randomization ensures that school-related and seasonal variations in sleep (e.g., school days vs. weekends, holiday periods) are balanced across study arms.

Only after the IC, adolescents are interviewed by the trial physician to check inclusion and exclusion criteria through self-report. To verify that all participants meet the DSM-5 criteria for chronic insomnia, the diagnosis will be confirmed by a physician using a structured clinical interview. During the screening for eligibility, parents are asked to support their children in reporting relevant medical data to ensure completeness. Concerning comorbidities, only confirmed diagnosis are considered. When all criteria are met, the person is included as a participant and informed about the eligibility by the physician directly.

Sociodemographic data is collected by the investigator and participants are asked to fill out the baseline questionnaires online (SecuTrial; link sent via chat of BBB). Upon completion of all baseline assessments, the participant is assigned to IG or CG (for randomization procedure see above). Participants are informed about the allocation directly and receive their personal code for *somnio junior* (IG) or the flyer with advice to sleep hygiene (CG) via e-mail. All of these steps are undertaken while still being in the video conference together, to ensure, participants receive all the information they need to proceed with the clinical trial without immediate assistance.

At 6- and 12-weeks post-randomization, the midpoint and post visits are conducted via BBB as well. To reduce bias due to the investigator knowing the group allocation, a standardized interview protocol is provided for the midpoint- and post-visit (Additional Files S5, S6). Investigators are instructed to strictly follow the protocol. While educating on study-specific information and screening for eligibility is done by a physician, psychologists will conduct the baseline assessment, initiates the randomization process and conduct midpoint and post visits. At the midpoint and post visits, investigators have knowledge of both baseline clinical information and allocation status. During these visits, adverse events are inquired. These also comprise worsening of e.g., affective symptoms. Further, changes in medication or psychotherapy and the periods during which school holidays took place are recorded. Questionnaires are filled out by the participants directly via the EDC (SecuTrial). At the post visit, after participants have filled out the t2-questionnaires, participants are asked an open-ended question about any additional strategies they used to improve their sleep outside the study intervention; responses will be categorized and summarized using descriptive frequency counts. Further, a semi-structured interview is conducted with participants of the IG to assess their attitude towards the digital intervention and to monitor participant adherence and engagement. The medical report is discussed to evaluate the individual development of different sleep-related parameters. All source data (e.g., protocols of visits, etc.) are digitalized and saved in pseudonymized form. Participants are financially compensated for their time by a 30€ voucher (10€ for each t0, t1 and t2 assessment).

### 2.6 Safety

The likelihood of serious adverse events (SAEs) occurring due to treatment is low since neither CBT-I nor sleep hygiene advice have been reported to cause them. We define SAEs as any untoward medical occurrence that either: (a) results in death, (b) is life- threatening, (c) requires inpatient hospitalisation or prolongation of existing hospitalisation, (d) results in persistent or significant disability/incapacity or (d) consists of a congenital anomaly or birth defect. Participants are asked for adverse events in the t1- and t2-visit and are further encouraged to inform the investigator at any time if any adverse event occurs. During the intervention period of the clinical trial, a monitor will review the study at least three times. Further visits will be added, if any deviations occur.

### 2.7 Measures

In addition to demographic measures (age, gender, education, comorbidities, sleep environment, duration of illness), data on the primary and secondary outcomes is raised through questionnaires. An overview is given in Table 2.

**Table 2:** Outcome measures.

	Outcome parameter	Questionnaire	Author
Primary endpoint	Insomnia severity	Insomnia Severity Index (ISI)	(46)
Secondary endpoints	Depressive symptoms	Patient Health Questionnaire-9 (PHQ-9)	(47)
	Anxiety symptoms	Generalized Anxiety Disorder (GAD-7)	(48)
	Daytime sleepiness	Stanford Sleepiness Scale (SSS)	(49)
	Health-related quality of life	Health-related quality of life in children and adolescents (KINDL)	(50)

#### 2.7.1 Primary endpoint: insomnia severity

The German translation of the Insomnia Severity Index (ISI), a 7-item questionnaire, is used because of its good psychometric properties, as a high construct validity (correlation with Pittsburgh Sleep Quality Index; PSQI r = 0.79) and high reliability (test-retest analysis r = 0.78). It is recommended using the ISI as a screening tool in research and it is adequately quantifying changes during insomnia therapy (46). The use of the ISI is approved by MapiTrust and the translation was supervised by ICON.

For the primary outcome (i.e., insomnia severity) a responder analysis of the Minimal Clinically Important Difference (MCID) for the Insomnia Severity Index [change in the ISI-score of ≥8 (51);] is performed. This is examined for group differences.

#### 2.7.2 Secondary endpoints

Measuring depressive symptoms, the Patient Health Questionnaire 9 (PHQ-9) is widely used and well validated. It is used here for its ability to detect changes in depressive symptoms over time (47). The Generalized Anxiety Disorder (GAD-7) is a short self-report questionnaire on symptoms of anxiety. Evidence from measuring subjects of the general population support validity and reliability of this instrument (48). For measuring subjective changes in daytime sleepiness, the Stanford Sleepiness Scale (SSS) is commonly used. It was shown to be sensitive to discrete changes in sleepiness and correlated with objective measures of vigilance (49). While originally validated in adults, the SSS has also been applied in research involving adolescents, including studies assessing sleepiness in school-aged populations and clinical trial contexts (52, 53). Its brief, momentary assessment format makes it feasible for repeated measurements in digital interventions targeting adolescents. Finally, for measuring the generic quality of life, the questionnaire “Health-related quality of life in children and adolescents” (Fragebogen zur Erfassung der gesundheitsbezogenen Lebensqualität bei Kindern und Jugendlichen; KINDL) is used. Results of psychometric testing of children with chronic illness and matched healthy children showed that the German KINDL is a practical, valid and reliable instrument (50).

#### 2.7.3 Exploratory analysis

Exploratory analysis is performed to assess the number of adverse events (AE) in both groups. We will compare the number of specified adverse events between the groups descriptively by reporting these by randomised group. Since school-related and seasonal variations (e.g., school days vs. weekends, holiday periods) are controlled for by randomization to account for such systematic errors, and the randomization procedure ensures that no more than 2–4 participants are assigned consecutively to either group, larger block formations are excluded. To avoid confounding or account for it appropriately, we will analyze whether the holiday status systematically differs between groups at the 6- and 12-week-post-randomization-assessments. If such a difference exists, a sensitivity analysis should be considered.

As part of the sleep diary, participants of the IG are asked to fill out details about their daytime activity (incl. caffeine or alcohol consumption) and their sleep behavior (time in bed, time they fell asleep) throughout the entire intervention by using the diary function of the application. This data will be analyzed exploratively with regard to sleep efficiency, total sleep time, time in bed, sleep latency, and wake-time after sleep onset in the IG only. Further, duration and frequency of application-usage will be analyzed. A brief semi-structured interview will be conducted with all intervention-group participants at the post-intervention visit (week 12) to assess their attitude towards the digital intervention and to monitor participant adherence and engagement. Interviews will be audio-recorded and transcribed verbatim. Data will be analyzed using reflexive thematic analysis following Braun & Clarke's established phases (familiarization, coding, theme development, refinement, and reporting). Two trained researchers will independently code transcripts, meet to consolidate the coding framework, and iteratively develop themes. Rigor will be ensured through independent coding, resolution of discrepancies via discussion, maintenance of coding logs and analytic memos, and regular peer debriefing. The qualitative findings will contextualize user experiences with the digital intervention. The measures participants have taken for improving their sleep will be analyzed on group differences using a poisson model. Finally, strategies for improving sleep in both groups will be collected and analyzed.

### 2.8 Statistical analysis

The primary statistical analysis will follow the intention-to-treat (ITT) principle. Efforts will be made to collect complete follow-up data for all participants to facilitate a comprehensive ITT analysis. However, missing data may occur due to withdrawals, loss to follow-up, or incomplete responses to some questionnaire items. Trial results will be presented as comparative summary statistics with 95% confidence intervals (CIs), and all tests will use a two-sided significance level of 5%. The study results will be reported in accordance with the Consolidated Standards of Reporting Trials (CONSORT) guidelines (54). A mixed effect linear model based on an unstructured covariance matrix will be fitted to the primary outcome data (ISI at 12 weeks), utilising 6- and 12-week timepoints. Participant will be included as random effects. Fixed effects will include randomized group, baseline ISI score, group, time and a time by randomized group interaction term to allow estimation of treatment effect at each timepoint. Missing data will be reported, including any available reasons for the missingness, and the patterns of missing data will be examined. The mixed-effects model inherently accounts for data assumed to be missing at random. Standard residual diagnostics will be conducted to evaluate the model's suitability. If the assumptions are found to be violated, alternative non-parametric methods will be considered for the primary analysis. Continuous secondary outcomes with only two time point measures (baseline and 12-weeks) will be analyzed with ANCOVA models, adjusting for baseline variable. The estimated difference between the groups after 12 weeks will be derived using a linear contrast statement from the ANCOVA model. Missing data, assuming confirmation of the “Missing at Random” (MAR) condition, will be estimated using multiple imputation. Subgroup analyses will be conducted based on gender (female, male). Secondary count outcomes (e.g., number of responders, number of side effects, number of measures to improve sleep) will be analyzed using Chi-Square tests. For the primary outcome, insomnia severity, a responder analysis will be conducted based on the Minimal Clinically Important Difference (MCID) for the Insomnia Severity Index (ISI), defined as a change in ISI ≥ 8. This analysis will examine differences between groups. Similarly, responder analyses will be conducted for depression [change in PHQ-9 ≥ 5 (47);] and anxiety measures [change in GAD-7 ≥ 3 (55);]. All outcomes were prespecified and are known to be correlated; therefore, consistent with methodological recommendations (56–58), no formal multiplicity adjustment will be applied.

The primary outcome measure is insomnia severity 12 weeks post-randomization. Assuming that this outcome follows a normally distributed population and that linear mixed models rely on pairwise comparisons, the hypothesis will be tested using a two-sided t-test for independent samples within a parallel-group design. To detect a statistically significant group difference with a Standardized Mean Difference (SMD) of 1.07 [the lower confidence interval of the within-group effect size reported in a prior study on the efficacy of digital CBT-I in adolescents using the Insomnia Severity Index (59);] with a power of 95% and an alpha level of 0.05, 24 participants per group are required [calculated using G*Power (60);]. Considering an anticipated dropout rate of 27% [based on (59)], a total of 66 adolescents should be randomized for the study.

## 3 Discussion

Despite the high prevalence of insomnia in adolescents and the wide range of associated problems, there is still no sufficient and adequate treatment for this target group. Although CBT-I as method of first choice is well validated (22, 23), too few children and adolescents receive access to this intervention (29). Tackling this care gap, digital interventions implementing CBT-I are evaluated in different studies (61–63) and meta-analyses reveal promising prospects of these interventions (31, 40, 64). De Bruin and colleagues (34) suggest that dCBT-I is a more cost-efficient therapy option and effects are comparable to CBT-I in a group setting.

If proven efficacious, the dCBT-I *somnio junior* aims to provide an economic and guideline-based treatment for adolescents suffering from insomnia. A strength of the current study is the clearly defined target population. While existing literature often includes samples ranging from general sleep problems to full insomnia symptoms—resulting in challenges for interpretation and comparability—the present study is based on a clinical diagnosis of insomnia made by a physician, according to DSM-V criteria. Therefore, results of this study can be directly applied to insomnia requiring treatment. With a bidirectional relationship between insomnia and mental disorders as depression, anxiety disorder (19) or other social-emotional and behavioral problems (14, 15), treating insomnia might also positively affect associated problems (65) as it was found to be a significant predictor for mental disorders as depression and anxiety (66). One possible mechanism connecting insomnia and mental health issues might be a compromised emotional regulation, which was found in people with sleep deprivation. Problems with regulation of emotion is a common underlying factor of psychiatric disorders (67). Indeed, next to symptoms of mental disorders, sleep deprivation is associated with emotional reactivity (68) and impulse control (17). Further, decreased sleep duration is a risk factor for several physical health concerns, including functional cortical alterations and systemic inflammation, hypertension, coronary artery disease, diabetes or obesity, and increased all-cause mortality (1, 12, 13). Thus, early treatment of insomnia might not only prevent chronification of insomnia symptoms, but might also have a preventative effect, so that subsequent disorders are less likely to occur. This could also be relevant for adolescents waiting for a psychotherapeutic treatment. The clear influence of sleep on physical health further undermines the importance of adequate treatment.

Accounting for these proposed associations, this study will evaluate both effects of the dCBT-I *somnio junior* on insomnia severity and on the related measures (daytime sleepiness, depression, anxiety and health-related quality of life). If the study shows the proposed effects *somnio junior* can be implemented as an economic, theory- and guideline-based intervention for the treatment of insomnia in adolescents.

### 3.1 Limitations

Due to the lack of a follow-up assessment, we will not be able to make assumptions on long-term effects of the application *somnio junior*. Since in most studies on dCBT-I, there were no changes from post- to follow-up assessment, we can assume, that this will be the case in the present study as well. However, future studies should evaluate long-term effects. Another limitation is that the intervention (interactive dCBT-I app) is contrasted with a low-intensity control (sleep hygiene flyer), resulting in unequal engagement and participant contact. This may introduce expectancy or attention effects that could contribute to observed differences. However, the comparator reflects minimal-care conditions typical of routine practice and avoids introducing additional active components. Furthermore, this study relies exclusively on validated self-report measures and sleep diaries. While the inclusion of objective monitoring (e.g., actigraphy) would have allowed for additional characterization of sleep–wake patterns and subjective–objective concordance, this was beyond the scope of the present study, which focused on perceived sleep difficulties and related distress—core features of insomnia as defined by the DSM-5. Future research may incorporate objective measures to address complementary questions regarding sleep physiology and circadian patterning. Lastly, we have planned exploratory analyses that are based on sleep diary data collected within the digital intervention only. As completion of the sleep diary is integrated into the application and forms part of the intervention, participants in the control group do not complete corresponding diary entries. Consequently, these data cannot be compared between groups, which substantially limits the interpretability of the exploratory analyses. Despite this constraint, the study is designed to provide meaningful evidence on whether dCBT-I *somnio junior* is effective in reducing insomnia symptoms and related outcomes in adolescents.

### 3.2 Further directions

If proven efficacious, the dCBT-I *somnio junior* provides an economic and guideline-based treatment for adolescents struggling with insomnia. In this case, *somnio junior* will be implemented as a digital health application (Digitale Gesundheitsanwendung; DiGA) and practitioners will be able to prescribe this DiGA for adolescents struggling with insomnia. Future studies should evaluate long-term effects and effects on objective sleep parameters. Next to the application for adolescents, a version of the dCBT-I tailored for parents supporting their children struggling with insomnia could further enhance the effectiveness of the intervention.

### 3.3 Trial status

In progress, recruiting.

## Supplementary material

Additional File 1 Completed SPIRIT checklist of recommended items to address in a clinical trial protocol (PDF 263 kb).

Additional File 2 Consent form for adolescents (PDF 484 kb).

Additional File 3 Consent form for custodians (PDF 518 kb).

Additional File 4 Flyer for the CG “Your Top 10 Rules for better sleep” in German (PDF 1.075 kb).

Additional File 5 Standardized interview protocol for the midpoint-visit (PDF 271 kb).

Additional File 6 Standardized interview protocol for the post-visit (PDF 326 kb).
