# Supplementary material for: Digital treatment for insomnia in adolescents: study protocol for a randomized controlled trial comparing digital cognitive behavioral therapy for insomnia to sleep hygiene
Source: Front Child Adolesc Psychiatry. 2026 May 1;5:1686491. doi: 10.3389/frcha.2026.1686491 (PMC13176156; doi:10.3389/frcha.2026.1686491)
Supplement: Additional File 1 — Completed SPIRIT checklist of recommended items to address in a clinical trial protocol (PDF 263 kb). [file Datasheet1.pdf]

Zentrum für Integrative Psychiatrie

Institut für Kinder- und Jugendpsychiatrie
Zentrum für Integrative Psychiatrie ZIP gGmbH
Universitätsklinikum Schleswig-Holstein, Campus Kiel
Niemannsweg 147, 24105 Kiel

Campus Kiel

Klinik für Psychiatrie und Psychotherapie
Direktorin Prof. Dr. Kamila Jauch-Chara

Klinik für Psychosomatik und Psychotherapie
Direktorin Prof. Dr. Kamila Jauch-Chara

Klinik für Psychiatrie, Psychotherapie und Psychosomatik des Kindes- und Jugendalters
Chefarzt Dr. Manuel Tobias Munz

Ambulanzzentrum
Direktorin Dr. Sibylle Wilms

Institut für Sexualmedizin und Forensische Psychiatrie und Psychotherapie Direktor Prof. Dr. Christian Huchzermeier

Institut für Kinder- und Jugendpsychiatrie
Direktorin Prof. Dr. Dr. Lioba Baving

Trauma-Ambulanz Flucht und Migration

SH Schleswig-Holstein Ministerium für Soziales, Gesundheit, Wissenschaft und Gleichstellung

www.zip.uksh.de

Ansprechpartner: Fee Meschkat
Tel.: 0431 500-98354
E-Mail: studie.somnio.kiel@uksh.de

Studieninformationen für Jugendliche (14-17 Jahre)

**Prüfstelle:**	Institut für Kinder Jugendpsychiatrie<br>Zentrum für Integrative Psychiatrie ZIP gGmbH<br>Niemannsweg 147<br>24105 Kiel<br>Tel.: 0431 500 98341<br>studie.somnio.kiel@uksh.de
**Prüfer:**	Prof. Dr. Alexander Prehn-Kristensen
**Prüfärztin/Prüfarzt:**	Telke Schoone
**EUDAMED-Nr.:**	CIV-24-05-046919
**Sponsor:**	mementor DE GmbH

„Eine randomisierte, kontrollierte klinische Prüfung zur Untersuchung der Wirksamkeit der digitalen Kognitiven Verhaltenstherapie *somnio junior* zur Reduzierung der Insomniesymptome bei Jugendlichen mit Insomnie nach dreimonatiger Nutzung“

Liebe Teilnehmerin, lieber Teilnehmer,

danke, dass Du Dich für unsere klinische Prüfung interessierst. Bevor Du Dich entscheidest mitzumachen, solltest Du wissen, was bei einer Teilnahme auf Dich zukommt. Auf den folgenden Seiten

erklären wir Dir, warum wir diese Prüfung machen und wie wir sie machen. Bitte lies Dir diese Informationen gut durch. Wenn Du Fragen hast oder etwas nicht verstehst, frag uns gerne! Auch wenn Dir später noch Fragen einfallen, darfst Du sie jederzeit stellen. Klinische Prüfungen sind notwendig, um Erkenntnisse über Medizinprodukte, wie z.B. auch die App „somnio junior“, zu gewinnen oder zu erweitern. Bevor neue Medizinprodukte zugelassen und verkauft werden dürfen, schreibt das Gesetz für Medizinprodukte vor, dass sie klinisch geprüft werden müssen. Die klinische Prüfung, die wir Dir hier vorstellen, wurde – wie es das Gesetz verlangt – von der zuständigen Ethikkommission zustimmend bewertet und von der zuständigen Behörde genehmigt. Diese klinische Prüfung wird im Institut für Kinder- und Jugendpsychiatrie am Zentrum für Integrative Psychiatrie ZIP gGmbH, Universitätsklinikum Schleswig-Holstein (UKSH), in Kiel durchgeführt. Insgesamt sollen ungefähr 66 Jugendliche zwischen 14 und 17 Jahren daran teilnehmen. Die Prüfung findet unter der Leitung von Herrn Prof. Dr. Alexander Prehn-Kristensen statt, welcher am Institut für Kinder- und Jugendpsychiatrie, UKSH Campus Kiel, wissenschaftlich tätig ist. Die Prüfung wird durch die Firma mementor DE GmbH gefördert, dem Hersteller der App „somnio junior“. Deine Teilnahme an dieser klinischen Prüfung ist freiwillig. Du wirst in diese Prüfung also nur dann einbezogen, wenn Du und Deine Sorgeberechtigten dazu schriftlich Eure Einwilligung erklären. Wenn Du nicht an der klinischen Prüfung teilnehmen oder später aus ihr ausscheiden möchtest, erwachsen Dir daraus keine Nachteile.

Informationen zum Ablauf der klinischen Prüfung

Warum wird diese klinische Prüfung durchgeführt?

Probleme mit dem Schlaf wirken sich schnell auf unser Wohlbefinden aus. Schlafprobleme können unterschiedlich aussehen. Manchmal gehen einem abends noch viele Gedanken durch den Kopf, so dass man nicht einschlafen kann; manchmal wacht man nachts auf und kann nicht wieder einschlafen, oder ist tagsüber sehr müde, kann aber abends trotzdem nicht einschlafen. Zur Behandlung von Ein- und Durchschlafstörungen wird in der Regel die sogenannte kognitive Verhaltenstherapie zur Behandlung der Insomnie (KVT-I) angewandt. Das ist eine Behandlung, bei der über die bewusste Veränderung von Verhaltensweisen, Denkmustern und Gefühlsbewertungen versucht wird, die Insomnie (Ein- und Durchschlafstörungen) zu bekämpfen. Hierfür trifft man sich in der Regel mehrmals mit einer Psychotherapeutin oder einem Psychotherapeuten in einer Praxis. Nun hat die Firma mementor DE GmbH eine App entwickelt, die im Prinzip genau dieselben Inhalte und Übungen vorgibt, wie die KVT-I in einer Psychotherapiepraxis. Die App heißt „somnio“. Innerhalb einer klinischen Prüfung an Erwachsenen hat sich gezeigt, dass sich „somnio“ zur Behandlung von Ein- und Durchschlafstörungen bei Erwachsenen eignet. Mittlerweile kann eine Ärztin oder ein Arzt die App auf

Rezept verschreiben. Deswegen sprechen wir bei „somnio“ auch von einer sogenannten „Digitalen Gesundheitsanwendung“ (DiGA). Da die App „somnio“ auf Erwachsene zugeschnitten ist, wurde die App „somnio junior“ speziell für Jugendliche mit Ein- und Durchschlafstörung entwickelt. „Somnio junior“ ist aus verschiedenen Modulen zusammengesetzt, deren Inhalte sich an der KVT.I orientieren. Die einzelnen Module der App haben jeweils unterschiedliche Themen: das Schlaftagebuch (Dokumentation des Schlafverhaltens), Schlafwissen (Informationen über Schlafstörungen, Schlafzyklus und Schlafzeiten etc.), Schlafverhalten (Tipp zu Verhaltensweisen im Bett), Stimuluskontrolle (Tipps zu Verbesserungen der Schlafumgebung), Entspannungstechniken, Achtsamkeitsübungen (praktische Übungen) sowie Verkürzung der Zeit die wach im Bett verbracht wird. Hierbei wirst Du von einer animierten digitalen Schlafexpertin oder einem Schlafexperten begleitet. In Abbildung 1 kannst Du verschiedene Ausschnitte aus der App „somnio junior“ sehen. Die App „somnio junior" kann noch nicht von einer Ärztin oder einem Arzt verschrieben werden, da sie nicht im Verzeichnis für digitale Gesundheitsanwendungen gelistet ist.

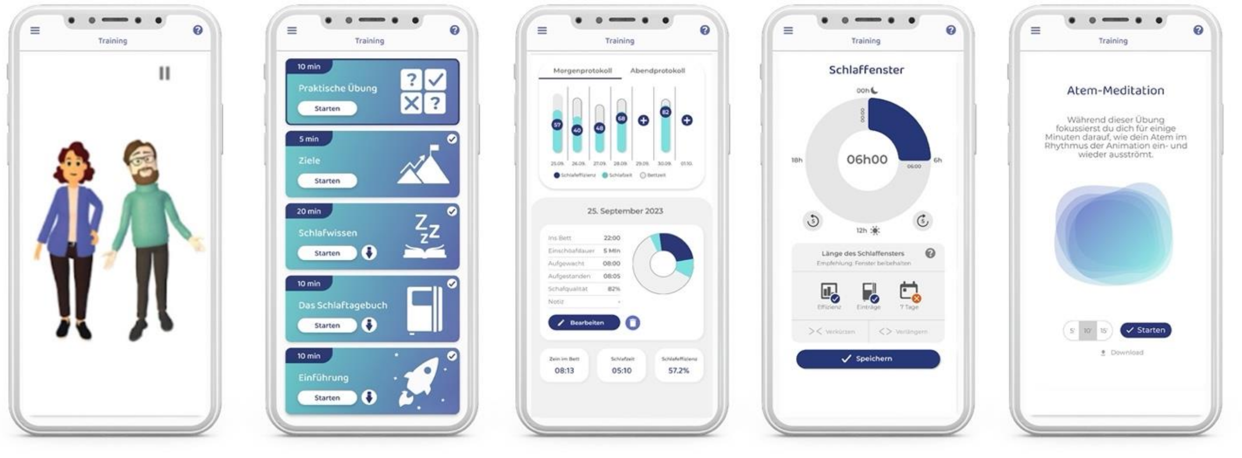

Abbildung 1. Darstellung der App „somnio junior“

Wir vom Institut für Kinder und Jugendpsychiatrie am Zentrum für Integrative Psychiatrie (ZIP gGmbH) Kiel möchten mit dieser Prüfung im Auftrag der Firma mementor DE GmbH herausfinden, ob „somnio junior“ bei Jugendlichen mit Ein- und Durchschlafstörungen helfen kann. Wenn sich die App in der Prüfung als wirksam erweisen, werden die Daten genutzt, um eine Aufnahme ins Verzeichnis für digitale Gesundheitsanwendungen zu unterstützen.

Wie wird die klinische Prüfung durchgeführt?

Die Prüfung findet digital statt, der Ablauf der Studie ist in Abbildung 2 dargestellt. So können deutschlandweit junge Menschen im Alter von 14 bis 17 Jahren mit einer Ein- und Durchschlafstörung teilnehmen. Die Prüfung besteht aus vier online-Terminen und der selbstständigen

Nutzung/Ausführung der App „somnio junior“ über 12 Wochen. Der genaue Ablauf wird hier grafisch dargestellt und wird im Verlauf beschrieben.

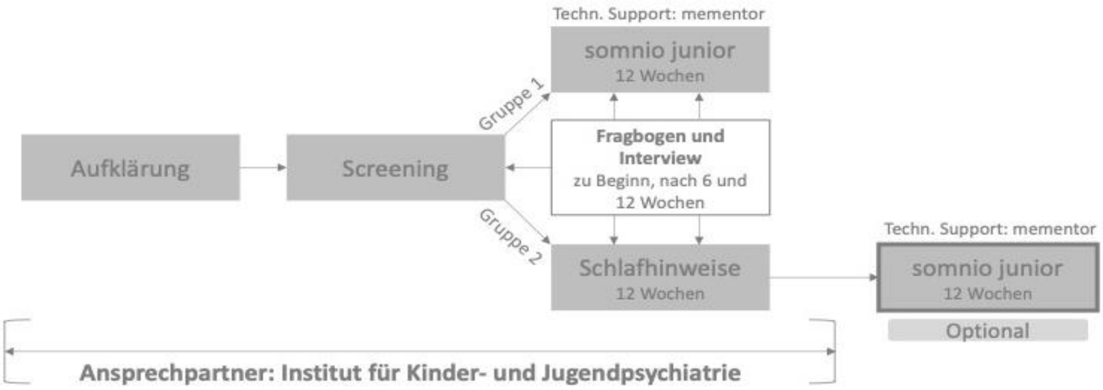

Abbildung 2: Ablauf der Prüfung.

Beim ersten online-Termin findet ein **Aufklärungsgespräch** statt, in welchem wir Dir und Deinen Sorgeberechtigten die Prüfung vorstellen. Für dieses Gespräch erhalten Du und Deine Sorgeberechtigten per E-Mail eine Einladung zu einem online-Termin, denn es ist wichtig, dass nicht nur Du, sondern auch alle Deine Sorgeberechtigten an dem Gespräch teilnehmen. Das online Aufklärungsgespräch findet auf einem gesicherten Server der Christian-Albrechts-Universität zu Kiel (CAU) statt, so dass niemand zuhören kann. Von unserer Seite nimmt an dem Gespräch eine Prüfärztin teil. In diesem Gespräch haben wir Zeit für alle Deine/Eure Fragen und besprechen, ob Du wirklich Lust hast, an der Prüfung teilzunehmen. Falls Du Dich für die Prüfung entscheidest, erhalten Du und Deine Sorgeberechtigten im Anschluss an das Aufklärungsgespräch die Einwilligungserklärungen für die Teilnahme an der Prüfung per E-Mail zugeschickt. Eine Voraussetzung für die Teilnahme ist nämlich, dass auch Deine Sorgeberechtigten damit einverstanden sind. Wenn Ihr Euch für eine Studienteilnahme entschieden habt, folgt einige Tage später der zweite online-Termin.

Beim zweiten online-Termin, dem **Screening**, treffen wir uns wieder im online-Besprechungsraum. An diesem Termin müssen Du und deine Sorgeberechtigten wieder gemeinsam teilnehmen, wenn Du unter 16 Jahren als bist. Falls Du bereits 16 Jahre oder älter bist können Du und deine Sorgeberechtigten gemeinsam entscheiden, ob Du allein oder Ihr gemeinsam an dem Termin teilnehmt. Von unserer Seite nehmen an dem Termin eine Psychologin und eine Prüfärztin teil. Bei diesem Screening stellen wir fest, ob Du bei der Prüfung mitmachen kannst, denn wir haben bestimmte Vorgaben, wer an der Prüfung teilnehmen kann und wer nicht. In diesem Gespräch stellen wir Dir einige Fragen zu Deiner Person, zu Deinem Schlaf und zu Deiner psychischen Gesundheit. Gleich im Anschluss

daran werden wir Dich bitten, online einen Fragebogen auszufüllen. Das Gespräch und die Beantwortung des online-Fragebogens werden insgesamt ca. 1 Stunde dauern. Diese Angaben helfen uns dabei zu entscheiden, ob Du an der Prüfung teilnehmen kannst. Bitte sei versichert, dass wir Deine Angaben streng vertraulich nach dem Datenschutzgesetz behandeln. Zum Thema Datenschutz findest Du alles Wichtige weiter unten in einem eigenen Absatz.

Wenn wir festgestellt haben, dass für Dich die Studienteilnahme möglich ist, dann wirst Du zufällig einer von zwei Gruppen zugeordnet: Die Gruppe 1 erhält sofort für 12 Wochen den vollen Zugang zu „somnio junior". Die Gruppe 2 erhält zunächst für 12 Wochen Hinweise zum Schlafverhalten, den vollen Zugang zu „somnio junior" erhält die Gruppe 2 jedoch erst im Anschluss an die Prüfung (nach 12 Wochen). Wir haben uns für diese zwei Gruppen entschieden, damit wir besser verstehen können, was innerhalb von 12 Wochen besser hilft: „somnio junior" oder die Hinweise zum Schlafverhalten. Das bedeutet aber auch, dass Du unabhängig von der Gruppeneinteilung in jedem Fall Zugang zur App „somnio junior" erhalten wirst.

Gruppe 1: Kurz nach dem Screening erhältst Du von uns per E-Mail einen Zugangscode zur App „somnio junior" zugeschickt. Anschließend lädst Du die App „somnio junior" auf Dein Smartphone, Tablet oder nutzt die Anwendung am Computer. Mit dem Zugangscode meldest Du Dich in der App an und folgst dort den weiteren Anweisungen. Hierzu gehört auch, dass Du den Allgemeinen Geschäftsbedingungen (AGB) der App somnio junior zustimmen musst. Den AGB müssen speziell deine Sorgeberechtigten zustimmen. Diese findest Du auch weiter unten in dieser Aufklärung. Wenn Du den AGB nicht zustimmen möchtest, dann ist das kein Problem. Allerdings kannst Du „somnio junior" dann nicht benutzen und auch nicht weiter an der Prüfung teilnehmen. Die App selbst beinhaltet mehrere Module, die Du zu Hause am Smartphone, Tablet oder Computer bearbeiten kannst. Die App nutzt Deine Ziele und Einträge, die Du in das Morgen- und Abendprotokoll einträgst, um auf Dich angepasst zu reagieren. Du erhältst Erinnerungsmails, wenn Du mal vergisst, das Morgen- und Abendprotokoll auszufüllen. Wir werden mithilfe der App über den gesamten Zeitraum von 12 Wochen Deine Nutzung der App und Dein Schlafverhalten beobachten. Falls Du Schwierigkeiten mit der App hast, kannst Du jederzeit Kontakt mit dem Support der Firma mementor aufnehmen und auch uns Bescheid geben. Weiter unten findest Du alle wichtigen Kontaktdaten. Falls es in der Zeit der Anwendung der Schlafhinweise zu gesundheitlichen Problemen irgendwelcher Art kommen sollte, melde diese bitte direkt an uns. Diese gesundheitlichen Probleme können direkt mit dem Schlaf zusammenhängen oder auch nur ein einfacher Schnupfen sein, bitte berichte uns alle gesundheitlichen Probleme. Wir nehmen nach ca. sechs Wochen Kontakt zu Dir auf, um Dir ein paar Fragen über Deine letzten sechs Wochen zu stellen (**Zwischen-Interview**) und Dich einen **online**-**Fragebogen** ausfüllen zu lassen. Wenn auch danach weiterhin alles gut läuft, dann beendest Du nach 12 Wochen die App und wir melden uns

wieder bei Dir, um das **Abschluss-Interview** durchzuführen und Dich zum letzten Mal einen Fragebogen ausfüllen zu lassen.

Gruppe 2: Falls Du zur Gruppe 2 gehörst, erhältst Du von uns eine Infobroschüre als PDF per E-Mail zugeschickt. In der Infobroschüre findest Du Hinweise, wie Du Deine Schlafumgebung und Dein Schlafverhalten verbessern kannst. Nach sechs und nach 12 Wochen melden wir uns, um Dir Fragen zu Dir und Deinen letzten sechs Wochen zu stellen (**Zwischen**-**Interview**) und jeweils einen **online-Fragebogen** ausfüllen zu lassen. Falls es in der Zeit der Anwendung der Schlafhinweise zu gesundheitlichen Problemen irgendwelcher Art kommen sollte, melde diese bitte direkt an uns. Diese gesundheitlichen Probleme können direkt mit dem Schlaf zusammenhängen oder auch nur ein einfacher Schnupfen sein, bitte berichte uns alle gesundheitlichen Probleme. Im Anschluss an die Studie (nach 12 Woche bzw. im Anschluss an die Abschlusserhebung) erhälst Du den Zugangscode zur App „somnio junior". Wir senden Dir dann einen Zugangscode zur App „somnio junior", mit dem Du die App für Dich für 12 weitere Wochen freischalten und nutzen kannst.

Welchen Nutzen hast Du von der Teilnahme an der klinischen Prüfung?

Wir hoffen sehr, dass Dir die App oder auch die Hinweise zum Schlafverhalten helfen, Deinen Schlaf zu verbessern. Jedoch können wir dies nicht mit Sicherheit versprechen. In jedem Fall hilfst Du uns damit zu verstehen, ob die App den Schlaf verbessern kann.

Welche Risiken sind mit der Teilnahme an der klinischen Prüfung verbunden?

Auch wenn wir davon überzeugt sind, dass Dir die Teilnahme an der Prüfung nicht schadet, kann es doch zu unerwünschten Ereignissen kommen. Die intensive Beschäftigung mit Deinem Schlaf kann zeitweise Dein Müdigkeitsgefühl verstärken. Einige der Übungen bringen vielleicht Deinen Tagesablauf durcheinander, sodass Du Dich auf einmal noch müder fühlst. Dies alles kann dazu führen, dass sich Deine Stimmung und Deine Leistungen in Schule, Ausbildung oder Beruf verschlechtern. Es könnte aufgrund vermehrter Müdigkeit auch zu Unfällen kommen. Wir bitten Dich, diese unerwünschten Veränderungen gut zu beobachten und diese dem Studienteam direkt mitzuteilen. Natürlich kannst Du auch jederzeit mit der Prüfung aufhören, wenn es Dir nicht mehr gefällt. Das haben wir unten nochmal ausführlicher beschrieben. Für die Nutzung der App „somnio junior" ist eine Internetverbindung über WLAN oder mobile Daten erforderlich. Es ist jedoch nicht notwendig, dass das Gerät dauerhaft mit dem Internet verbunden ist, sondern nur während der Nutzung der App. Somnio junior kann auch browserbasiert, ausschließlich am Computer durchgeführt werden. Eventuell anfallende Roaming-Gebühren während eines Auslandsaufenthalts müssen selbst getragen werden. Bitte prüfe gemeinsam mit Deinen Sorgeberechtigten Deinen Mobilfunktarif, um unerwartete Kosten zu vermeiden.

Wer kann an der Prüfung teilnehmen?

- Personen, die unter Ein- oder Durchschlafproblemen leiden
- Personen, die zwischen 14 und17 Jahren alt sind
- Personen mit Zugang zu einem Tablet/Smartphone mit einem Internetzugang
- Personen, welche die deutsche Sprache sprechen und lesen können

Was sind Ausschlusskriterien für die Teilnahme an der Studie?

- Akute Suizidalität
- Epilepsie
- Bipolare Störung
- körperliche Erkrankungen, die einen maßgeblichen Einfluss auf den Schlaf haben können wie bei akuten oder chronischen Hauterkrankungen (insbesondere bei vermehrtem Juckreiz z.B. bei Neurodermitis, Läusebefall und Krätze), Schmerz- oder Atemwegserkrankungen
- Vollstationäre psychiatrische Krisenintervention innerhalb der letzten vier Wochen
- Suchterkrankungen inklusive Medienbezogene Störung
- Teilnahme an einer anderen klinischen Prüfung

Schwangerschaft

Falls du während der Studie schwanger wirst, melde dich bitte sofort beim Studienzentrum. Du darfst dann nicht weiter an der Studie teilnehmen.

Bekommst Du etwas für die Teilnahme an der klinischen Prüfung?

Ja, Du erhältst für jeden der drei Terminblöcke (Screening, Termin nach sechs und 12 Wochen) einen 10 Euro Wertgutschein. Du erhältst, wenn Du an allen drei Terminen teilnimmst, so insgesamt einen Wertgutschein in Höhe von 30 Euro. Den Wertgutschein erhältst Du nach Abschluss der Prüfung.

Freiwilligkeit:

Bei unserer Prüfung machst Du freiwillig mit. Du entscheidest, ob Du mit der Prüfung beginnen möchtest und wie lange Du dabeibleibst. Auch wenn Deine Sorgeberechtigten einverstanden sind, kannst Du frei entscheiden, „nein“ zu sagen. Du hast keinen Nachteil, wenn Du nicht mitmachen willst. Du kannst auch während der Prüfung jederzeit aufhören. Falls Du Dich entscheidest nicht mehr an der Prüfung teilzunehmen werden wir versuchen, Dich einmalig per Telefon oder E-Mail zu kontaktieren,

um Dich nach dem Grund für Deine Entscheidung zu fragen. Du musst uns nicht sagen warum. Du hast auch keinen Nachteil dadurch.

Versicherungsschutz:

Du bist während der Prüfung gegen eventuelle Gesundheitsschäden durch Deine Studienteilnahme versichert. Nähere Informationen dazu haben Deine Eltern erhalten. Das betrifft auch die Frage, was geschehen muss, wenn Du den Verdacht hast, dass die Studie bei Dir einen Gesundheitsschaden verursacht haben könnte.

Informationen zum Datenschutz:

Was geschieht mit den Daten, die über Dich erhoben werden? Der Datenschutz innerhalb der Prüfung ist durch das Datenschutzgesetz (die Europäische Datenschutz-Grundverordnung) genau geregelt, nähere Informationen dazu haben Deine Sorgeberechtigten erhalten. Zum leichteren Verständnis haben wir Dir hier die wichtigsten Punkte zusammengefasst: Wir erfahren während dieser Prüfung viel über Dich, beispielweise schreiben wir uns Deinen Namen und Deinen Geburtstag auf. Diese Daten werden von uns besonders sorgfältig behandelt und geschützt. Wir dürfen Deine Daten nicht ohne Dein Einverständnis weitergeben. Wenn Du Deine Teilnahme an der Prüfung beenden möchtest, müssen die bereits gespeicherten Angaben über Dich und Deine Krankheit weiter gespeichert bleiben. Um Deine Identität zu schützen, werden wir Deinem Namen einen Code zu ordnen. Wir nutzen in unseren Unterlagen dann nur noch Deinen Code anstelle Deines Namens. Der Code besteht nur aus Zahlen und Buchstaben, niemand außer uns kann den Code Deinem Namen zuordnen. Die Liste mit der Zuordnung Deines Namens zu dem Code wird bei uns sicher in abschließbaren Datenschränken im Institut für Kinder- und Jugendpsychiatrie aufbewahrt. Im Anschluss an die Studie wird ein Bericht mit den Daten aus der Prüfung geschrieben. In dem Bericht erfährt niemand, dass Du bei der Prüfung mitgemacht hast. Wir können die Daten auch für andere Prüfungen nutzen, dafür bewahren wir die Daten manchmal länger als 10 Jahre auf.

Alexander Prehn-Kristensen und Fee Meschkat leiten diese Prüfung. Die beiden sind verantwortlich, dass sich alle Studienmitarbeiter an diese Regeln halten. Wenn wir uns nicht an die Regeln halten, kannst Du Dich über uns beschweren. Du findest eine Liste mit den Kontaktdaten am Ende dieses Dokumentes. Wir dürfen Deine Daten nur erfragen, speichern, nutzen und weitergeben, wenn Du es erlaubst, dafür gibt es die Einwilligungs-Erklärung zum Datenschutz. Du stimmst zu, wenn Du sie unterschreibst. Im Zusammenhang mit dieser Prüfung wirst Du die App „somnio junior“ verwenden, die Daten, die im Zusammenhang mit Deiner Nutzung entstehen, werden uns mit Hilfe Deines Codes weitergegeben. Deine Sorgeberechtigten und Du müssen, um die App zu nutzen, den Allgemeinen

Geschäftsbedingungen (AGB) zu stimmen, hier kannst Du Dir die AGBs und Deine Rechte im Rahmen der Datenschutzverordnung anschauen.

Allgemeine Geschäftsbedingungen der App „somnio junior“

Wir haben Dir die AGBs hier schon vorab zur Verfügung gestellt, damit Du sie Dir schon im Vorfeld anschauen kannst.

1 Allgemeines

- Du schließt eine Nutzungsvereinbarung mit der mementor DE GmbH (nachfolgend mementor) ab. Indem Du diese AGB akzeptierst, werden diese Vertragsbestandteil in Bezug auf die Nutzung des Online-Programms somnio junior.

2 Gegenstand

- Gegenstand dieser AGB ist die Nutzung des Online-Programms *somnio junior*. *somnio junior* bietet Dir Informationen und Übungen rund um das Thema Schlafstörungen, die auf wissenschaftlich geprüften, psychotherapeutischen Methoden basieren.
- Wir weisen ausdrücklich darauf hin, dass die Nutzung von *somnio junior* kein Ersatz für eine Diagnose oder eine ärztliche bzw. psychotherapeutische Behandlung ist.

3 Zugangsberechtigung

- Die Voraussetzung für die Nutzung von *somnio junior* ist eine Registrierung. Mit der Registrierung werden die AGB und die Datenschutzerklärung anerkannt. Wenn Du jünger als 18 Jahre alt bist, muss mindestens einer Deiner gesetzlichen Vertreter (in der Regel sind das Deine Eltern) schriftlich eingewilligt haben. mementor kann Dich jederzeit auffordern, einen Nachweis über Deine Identität, Dein Alter und/oder die Zustimmung Deiner gesetzlichen Vertreter vorzulegen. Die AGB treten in Kraft, sobald die Registrierung für *somnio junior* abgeschlossen wurde.
- Die Registrierung ist nur mit einem Lizenzcode möglich.

4 Deine Rechte

- Für die Nutzung von *somnio junior* erhältst Du das einfache, nicht ausschließliche und auf Dritte nicht übertragbare Nutzungsrecht. Ein Erwerb an den Inhalten ist damit nicht verbunden.
- *somnio junior* steht Dir während der Lizenzlaufzeit grundsätzlich durchgehend zur Verfügung. Aus technischen Gründen (z. B. wegen routinemäßiger oder erforderlicher Wartungsarbeiten) kann die Verfügbarkeit von *somnio junior* zeitweise eingeschränkt sein.

5 Deine Pflichten

- Für den Zugang zum Programm benötigst Du individuelle Anmeldedaten, für deren Geheimhaltung Du verantwortlich bist und deren Missbrauch Du zu verhindern hast. Bei Missbrauch jeglicher Art hast Du mementor hierüber zu informieren über die E-Mail-Adresse support@junior-somni.io. mementor ist bei einer Vertragsverletzung dazu berechtigt, den betroffenen Zugang zu sperren. Du haftest für einen von Dir zu vertretenden Missbrauch.
- Die für die Nutzung von *somnio junior* anfallenden Endgerätekosten (z.B. Laptop) und Telekommunikationsentgelte (z.B. Kosten für den Internetzugang) sind von Dir zu tragen. Für eine Nutzung außerhalb dieses Landes können entsprechende Roaming-Gebühren anfallen, die von Dir zu tragen sind.
- Erforderliche Software (z.B. Betriebssystem oder Internetbrowser), die für die Nutzung von *somnio junior* notwendig ist, ist von Dir zu installieren. Des Weiteren bist Du dafür verantwortlich, die technischen Voraussetzungen für den Zugang zu *somnio junior* zu schaffen, insbesondere hinsichtlich der eingesetzten Hardware, der Betriebssystemsoftware, der Verbindung zum Internet und der Browsersoftware.
- Im Falle der Weiterentwicklung von *somnio junior* bist Du dafür verantwortlich, die erforderlichen Anpassungen bei der von Dir eingesetzten Hard- und Software zu treffen (z.B. Aktualisierung des Browsers).
- Solltest Du dich aus irgendeinem Grund tagsüber sehr müde fühlen, solltest Du auf das Autofahren und auf das Bedienen von Maschinen verzichten.

6 Vertragsverletzungen

- Im Falle einer Vertragsverletzung Deinerseits ist mementor zur fristlosen Kündigung berechtigt. Schadensersatzansprüche bleiben hiervon unberührt

7 Haftungsausschluss

- mementor haftet bei Vorsatz und grober Fahrlässigkeit nach den gesetzlichen Bestimmungen. Bei leicht fahrlässig verursachten Verletzungen wesentlicher Vertragspflichten haftet mementor für den vorhersehbaren,

vertragstypischen Schaden. Wesentliche Vertragspflichten sind solche, die für die ordnungsgemäße Durchführung des Vertragszweckes von besonderer Bedeutung sind und auf deren Einhaltung der Kunde vertrauen durfte. Im Übrigen ist die Haftung für leichte Fahrlässigkeit ausgeschlossen

- Für von mementor nicht verschuldete Störungen innerhalb des Leitungsnetzes übernimmt die Firma keine Haftung.
- Die Haftung aufgrund zwingender gesetzlicher Vorschriften, einschließlich derjenigen des Produkthaftungsgesetzes, bleibt unberührt.
- Du bist für eine regelmäßige Sicherung Deiner Daten verantwortlich. Bei einem Datenverlust haftet mementor höchstens in Höhe des Aufwands, der zur Wiederherstellung der Sicherungen von deinen Daten notwendig ist.
- Die mementor-Apps bzw. unsere Webseite können auch Links oder Verweise auf Internet-Seiten Dritter enthalten, auf deren Inhalt wir keinen Einfluss haben. Deshalb übernehmen wir trotz sorgfältiger inhaltlicher Kontrolle keine Haftung für die Inhalte externer Links. Für den Inhalt der verlinkten Seiten sind ausschließlich deren Betreiber verantwortlich.
- Die individuellen Unterschiede unter allen Nutzer:innen und in jeder körperlichen Verfassung sind vielfältig. Die Gesundheit eines jedes Einzelnen hängt von einer Vielzahl verschiedener Faktoren ab. Auch der Erfolg eines durch mementor zur Verfügung gestellten digitalen Programms ist von verschiedenen Faktoren abhängig, auf die mementor keinen Einfluss hat. Folglich kann keine Garantie für den Erfolg eines App-Programms übernommen werden.
- Eine Haftung von mementor für die Richtigkeit des digitalen Programms ist ausgeschlossen, sofern Du falsche oder nicht vollständige Angaben, insbesondere zum Gesundheitszustand, zu Vorerkrankungen oder dem Vorliegen von Erkrankungen jeglicher Art gemacht hast oder es unterlassen hast, solche Angaben zu machen.
- Eine Haftung von mementor ist ausgeschlossen, sofern die Inhalte des Programms entgegen ihrer Zweckbestimmung zu einer Selbstdiagnose und/oder -behandlung herangezogen werden. Das Gleiche gilt, falls aufgrund der erteilten Informationen und Auskünfte die notwendige Konsultation einer Fachperson nicht stattfindet.

8 Urheberrecht

- Die Inhalte des Online-Programms *somnio junior* unterliegen dem Urheberrecht von mementor. Die Inhalte beruhen auf wissenschaftlich anerkannten psychologischen Methoden, die von psychologischen Expert:innen entwickelt worden sind.
- Du hast die Möglichkeit, Material und Informationen für den persönlichen Gebrauch herunterzuladen. Dieses Material ist ausschließlich zu Deiner eigenen Nutzung bestimmt und darf nicht an Dritte weitergegeben oder kommerziell verbreitet werden. Jede Verbreitung, Veränderung oder Reproduktion von Material und Informationen ist nicht gestattet.
- Bilder, Graphiken und das Layout von *somnio junior* sind Eigentum von mementor und dürfen ohne vorherige schriftliche Genehmigung nicht reproduziert oder verbreitet werden.

9 Aktualisierungen/Änderungen

- mementor behält sich vor, ohne vorherige Ankündigung Änderungen oder Ergänzungen der bereitgestellten Informationen vorzunehmen. Eine Haftung ergibt sich daraus nicht.

10 Schlussbestimmungen

- Es gilt deutsches Recht.
- Gerichtsstand für alle Streitigkeiten aus diesem Vertragsverhältnis ist Leipzig.
- Sollten einzelne Regelungen dieser AGB unwirksam oder anfechtbar sein, wird dadurch die Wirksamkeit der übrigen Regelungen nicht berührt.

11 Kontakt

- Du kannst uns über Email oder telefonisch kontaktieren: support@junior-somnio.io / 0341 97852812.

Wem kannst Du weitere Fragen stellen?

Wenn Du Fragen hast, dann melde Dich einfach beim Studienteam (Tel.: 0431 500 98345, Mail: studie.somnio.kiel@uksh.de). Sie beantworten gerne Deine Fragen im Zusammenhang mit der Prüfung. Bei technischen Fragen im Zusammenhang mit der Nutzung der App melde Dich gern beim Support-Team (Tel.: 0341 97852812, Mail: support@junior-somnio.io).

**Weitere Kontaktdaten**
**Studienverantwortliche**<br>Prof. Dr. Alexander Prehn-Kristensen<br>Stellvertretender Direktor<br>Institut für Kinder- und Jugendpsychiatrie<br>Zentrum für Integrative Psychiatrie ZIP gGmbH, Campus Kiel<br>Universitätsklinikum Schleswig-Holstein<br>Niemannsweg 147, 24105 Kiel<br>Tel.: 0431 500 98316<br>Mail: alexander.prehn-kristensen@uksh.de<br><br>M.Sc. Psych. Fee Meschkat<br>Wissenschaftliche Mitarbeiterin<br>Institut für Kinder- und Jugendpsychiatrie<br>Zentrum für Integrative Psychiatrie ZIP gGmbH, Campus Kiel<br>Universitätsklinikum Schleswig-Holstein<br>Niemannsweg 147, 24105 Kiel<br>Tel.: 0431 500 -98345<br>Mail: sarahfee.Meschkat@uksh.de
**mementor DE GmbH**<br>Kontaktdaten des Datenschutzbeauftragten<br>Ansprechpartner: Paul Schmude<br>Karl-Heine-Straße 15, 04229 Leipzig<br>Mail: dataprivacy@mementor.de<br><br>**Technischer Support**<br>Kontaktdaten vom Technischen Support<br>Tel.: 0341 97852812<br>Mail: support@junior-somnio.io
**Prüfärztin**<br>Telke Schoone<br>Klinik für Psychiatrie, Psychotherapie und Psychosomatik des Kindes- und Jugendalters<br>Zentrum für Integrative Psychiatrie ZIP gGmbH<br>Universitätsklinikum Schleswig-Holstein, Campus Kiel<br>Niemannsweg 147, 24105 Kiel<br>Telefon: 0431 500 98344<br>Mail: telke.schoone@uksh.de
**Empfänger Deiner personenbezogenen Daten**<br>Die Daten werden von uns (Institut für Kinder- und Jugendpsychiatrie am ZIP) verarbeitet.
**Datenschutzbeauftragte der Christian-Albrechts-Universität zu Kiel (CAU)**<br>Frau Stella Thoben<br>Tel.: 0431 880 3581<br>Mail: sthoben@uv.uni-kiel.de
**Landesbehörde Schleswig-Holstein**

Unabhängiges Landeszentrum für Datenschutz (ULD) Schleswig-Holstein
Ansprechpartnerin: Marit Hansen, Landesbeauftragte für Datenschutz Schleswig-Holstein
Holstenstraße 98, 24103 Kiel
Tel.: 0431 988 1200
Mail: mail@datenschutzzentrum.de

Vielen Dank, dass Du diese Information bis zum Ende durchgelesen hast.
Wir freuen uns auf Deine Fragen!

Prüfstelle:
Institut für Kinder- und Jugendpsychiatrie
Zentrum für Integrative Psychiatrie ZIP gGmbH
Universitätsklinikum Schleswig-Holstein, Campus Kiel
Niemannsweg 147, 24105 Kiel

EUDAMED-Nr. CIV-24-05-046919

Eine randomisierte, kontrollierte klinische Prüfung zur Untersuchung der Wirksamkeit der digitalen Kognitiven Verhaltenstherapie *somnio junior* zur Reduzierung der Insomniesymptome bei Jugendlichen mit Insomnie nach dreimonatiger Nutzung

Einwilligungserklärung

____

Vor- und Nachnamen Teilnehmer/Teilnehmerin in Druckbuchstaben

____

Geburtsdatum

Ich bin in einem persönlichen Gespräch durch den Prüfarzt/ die Prüfärztin

____

Name der Ärztin/ des Arztes

Voraussetzung für Deine Teilnahme ist, dass Du einverstanden bist. Wenn ja, bitten wir Dich, auf diesem Blatt zu unterschreiben. Du bestätigst uns damit, dass Du an der klinischen Prüfung teilnehmen möchtest und weißt, dass dies freiwillig ist, alle Deine Fragen zu Deiner Zufriedenheit beantwortet wurden und Du genügend Zeit hattest, Deine Teilnahme zu bedenken. Du kannst aber auch später zu jeder Zeit sagen, dass Du nicht mehr an der klinischen Prüfung teilnehmen möchtest, Dir werden daraus keine Nachteile entstehen.

Ich erkläre ich freiwillig bereit, an der oben genannten klinischen Prüfung teilzunehmen. Mit der Erhebung und Verarbeitung meiner Daten, wie in der mir ausgehändigten Informationsschrift beschrieben, bin ich einverstanden.

____

Datum, Unterschrift Teilnehmer/Teilnehmerin

Ich habe das Aufklärungsgespräch geführt, die Jugendlichen und ihre Sorgeberechtigten wurden von mir über den Inhalt und Ablauf der Prüfung sowie Vor- und Nachteile der Teilnahme informiert.

____

Name Prüfarzt/ Prüfärztin in Druckbuchstaben

____

Datum, Unterschrift Prüfarzt/ Prüfärztin

**Datenschutzrechtliche Einwilligung: Tonaufzeichnung**
Ich willige ein, dass im Rahmen dieser klinischen Studie digitale Tonaufzeichnungen von meinem Abschlussgespräch mit dem Studienteam gemacht werden. Ich habe verstanden, dass die Aufzeichnung optional ist und ich auch ohne meine Einwilligung in die Tonaufzeichung an der klinischen Studie teilnehmen kann.<br>☐ Ja ☐ nein<br>...<br>Datum, Unterschrift Teilnehmer/Teilnehmerin
