# Supplementary material for: Digital treatment for insomnia in adolescents: study protocol for a randomized controlled trial comparing digital cognitive behavioral therapy for insomnia to sleep hygiene
Source: Front Child Adolesc Psychiatry. 2026 May 1;5:1686491. doi: 10.3389/frcha.2026.1686491 (PMC13176156; doi:10.3389/frcha.2026.1686491)
Supplement: Additional File 2 — Consent form for adolescents (PDF 484 kb). [file Datasheet2.pdf]

Institut für Kinder- und Jugendpsychiatrie
Zentrum für Integrative Psychiatrie ZIP gGmbH
Universitätsklinikum Schleswig-Holstein, Campus Kiel
Niemannsweg 147, 24105 Kiel

Campus Kiel

Klinik für Psychiatrie und Psychotherapie
Direktorin Prof. Dr. Kamila Jauch-Chara

Klinik für Psychosomatik und Psychotherapie
Direktorin Prof. Dr. Kamila Jauch-Chara

Klinik für Psychiatrie, Psychotherapie und Psychosomatik des Kindes- und Jugendalters
Chefarzt Dr. Manuel Tobias Munz

Ambulanzzentrum
Direktorin Dr. Sibylle Wilms

Institut für Sexualmedizin und Forensische Psychiatrie und Psychotherapie Direktor Prof. Dr. Christian Huchzermeier

Institut für Kinder- und Jugendpsychiatrie
Direktorin Prof. Dr. Dr. Lioba Baving

Trauma-Ambulanz Flucht und Migration

Schleswig-Holstein
Ministerium für Soziales, Gesundheit, Wissenschaft und Gleichstellung

www.zip.uksh.de

Ansprechpartner: Beke Ralfs
Tel.: 0431 500-98354
E-Mail: studie.somnio.kiel@uksh.de

Studieninformationen für Sorgeberechtigte

Prüfstelle: Institut für Kinder Jugendpsychiatrie
Zentrum für Integrative Psychiatrie ZIP gGmbH
Niemannsweg 147
24105 Kiel
Tel.: 0431 500 98341
studie.somnio.kiel@uksh.de

Prüfer: Prof. Dr. Alexander Prehn-Kristensen

Prüfärztin/Prüfarzt: Telke Schoone

EUDAMED-Nr.: CIV-24-05-046919

Sponsor: mementor DE GmbH

„Eine randomisierte, kontrollierte klinische Prüfung zur Untersuchung der Wirksamkeit der digitalen Kognitiven Verhaltenstherapie *somnio junior* zur Reduzierung der Insomniesymptome bei Jugendlichen mit Insomnie nach dreimonatiger Nutzung“

Liebe Eltern,

die folgenden Seiten sollen Sie über unser Forschungsziel und den geplanten Ablauf der klinischen Prüfung informieren. Bevor Sie sich für eine Teilnahme Ihres Kindes an der Prüfung entscheiden, lesen Sie sich bitte dieses Informationsblatt gut durch. Bitte zögern Sie nicht, alle Punkte anzusprechen, die

Ihnen unklar sind. Klinische Prüfungen sind notwendig, um Erkenntnisse über Medizinprodukte, wie z.B. auch die App „somnio junior“, zu gewinnen oder zu erweitern. Bevor neue Medizinprodukte zugelassen und verkauft werden dürfen, schreibt das Gesetz für Medizinprodukte vor, dass sie klinisch geprüft werden müssen. Die klinische Prüfung, die wir Ihnen hier vorstellen, wurde – wie es das Gesetz verlangt – von der zuständigen Ethikkommission zustimmend bewertet und von der zuständigen Behörde genehmigt. Diese klinische Prüfung wird im Institut für Kinder- und Jugendpsychiatrie am Zentrum für Integrative Psychiatrie ZIP gGmbH, Universitätsklinikum Schleswig-Holstein (UKSH), in Kiel durchgeführt. Insgesamt sollen ungefähr 66 Jugendliche zwischen 14 und 17 Jahren daran teilnehmen. Die Prüfung findet unter der Leitung von Herrn Prof. Dr. Alexander Prehn-Kristensen statt, welcher am Institut für Kinder- und Jugendpsychiatrie, UKSH Campus Kiel, wissenschaftlich tätig ist. Die Prüfung wird durch die Firma mementor DE GmbH gefördert, die die App „somnio junior“ herstellt. Die Teilnahme Ihres Kindes an dieser klinischen Prüfung ist freiwillig. Ihr Kind wird in diese Prüfung nur dann einbezogen, wenn auch Sie als Sorgeberechtigte dazu schriftlich Ihre Einwilligung erklären. Sofern Ihr Kind nicht an der klinischen Prüfung teilnehmen oder später aus ihr ausscheiden möchte, erwachsen daraus keine Nachteile.

Informationen zum Ablauf der klinischen Prüfung

Warum wird diese klinische Prüfung durchgeführt?

Schlafprobleme können bereits im Kindes- und Jugendalter beginnen und sind häufig nicht nur ein Belastungsfaktor für die Betroffenen selbst, sondern wirken sich negativ auf das gesamte Familienleben aus. Eine häufige Form von Schlafproblemen sind Ein- und Durchschlafstörungen. Man spricht bei schweren und überdauernden Ein- und Durchschlafstörungen auch von einer Insomnie. Der damit verbundene Schlafmangel und die schlechtere Schlafqualität können zu weiteren Problemen wie Konzentrationsschwierigkeiten und Tagesschläfrigkeit führen. Zur Behandlung von Ein- und Durchschlafstörungen wendet man in der Regel die kognitive Verhaltenstherapie zur Behandlung der Insomnie (KVT-I) an. Das ist eine Behandlung, bei der über die Veränderung von Verhaltensweisen, Denkmustern und Gefühlsbewertungen versucht wird, die Insomnie zu bekämpfen. Die Behandlung besteht aus mehreren Sitzungen mit einer Psychotherapeutin oder einem Psychotherapeuten in einer Praxis. Die Firma mementor DE GmbH hat eine App entwickelt, die im Prinzip genau dieselben Inhalte und Übungen vorgibt wie auch die KVT-I in einer Psychotherapie. Die App heißt „somnio“ und wurde bereits in einer klinischen Prüfung an Erwachsenen auf ihre Wirksamkeit untersucht. Die Ergebnisse haben gezeigt, dass sich „somnio“ zur Behandlung von Ein- und Durchschlafstörungen bei Erwachsenen eignet. Eine Ärztin oder ein Arzt kann die App „somnio“ inzwischen auf Rezept verschreiben, da sie ins Verzeichnis für digitale Gesundheitsanwendungen aufgenommen wurde – daher sprechen wir bei

„somnio“ auch von einer „Digitalen Gesundheitsanwendung“. Als Ergänzung zur App „somnio“ hat die Firma mementor DE GmbH die App „somnio junior“, speziell für Jugendliche mit Ein- und Durchschlafstörungen, entwickelt. Somnio junior ist aus verschiedenen Modulen zusammengesetzt, deren Inhalte sich an der KVT-I orientieren. Die Bestandteile der App sind das Schlaftagebuch, Schlafwissen (Informationen über Schlafstörungen, Schlafzyklen, Schlafzeiten etc.), Schlafverhalten (Verhaltensweisen im Bett), Stimuluskontrolle (Verbesserung der Schlafumgebung), Entspannungstechniken, Achtsamkeit (praktische Übungen) sowie Bettzeitrestriktion zur Verbesserung des Schlafrhythmus (Verkürzung der Zeit, die wach im Bett verbracht wird). Hierbei werden die Jugendlichen je nach Präferenz durchgehend von einer animierten digitalen Schlafexpertin oder einem Schlafexperten begleitet. In Abbildung 1 sind verschiedene Ausschnitte aus der App somnio junior dargestellt. Die App „somnio junior“ kann nicht von einer Ärztin oder einem Arzt verschrieben werden, da sie nicht im Verzeichnis für digitale Gesundheitsanwendungen gelistet ist.

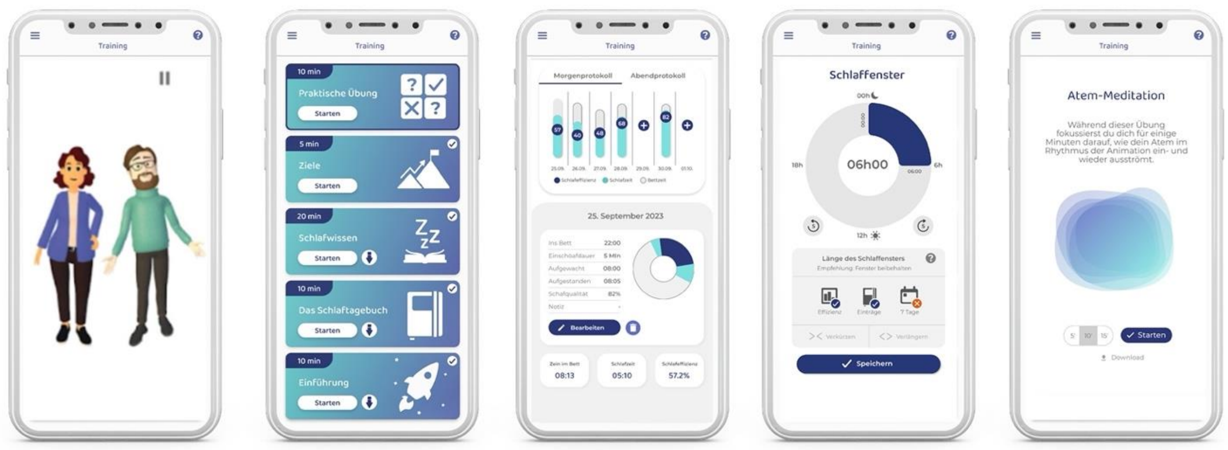

Abbildung 1. Darstellung der App „somnio junior“

Wir vom Institut für Kinder- und Jugendpsychiatrie am Zentrum für Integrative Psychiatrie (ZIP gGmbH) Kiel möchten im Auftrag der Firma mementor DE GmbH mit dieser Prüfung nun herausfinden, ob „somnio junior“ auch Jugendlichen mit Ein- und Durchschlafstörungen helfen kann. Wenn sich die App innerhalb der Prüfung als wirksam erweist, werden die Daten genutzt, um eine Aufnahme ins Verzeichnis für digitale Gesundheitsanwendungen zu unterstützen.

Wie wird die klinische Prüfung durchgeführt?

Die Prüfung wird vollständig digital durchgeführt, der Ablauf der Studie ist in Abbildung 2 dargestellt. So können deutschlandweit junge Menschen im Alter zwischen 14 und 17 Jahren mit einer Ein- und Durchschlafstörung teilnehmen. Die Prüfung besteht insgesamt aus vier online-Terminen und der selbständigen Nutzung/Ausführung der App „somnio junior“ über 12 Wochen. Im Weiteren erklären wir, was genau auf die Teilnehmenden zukommt.

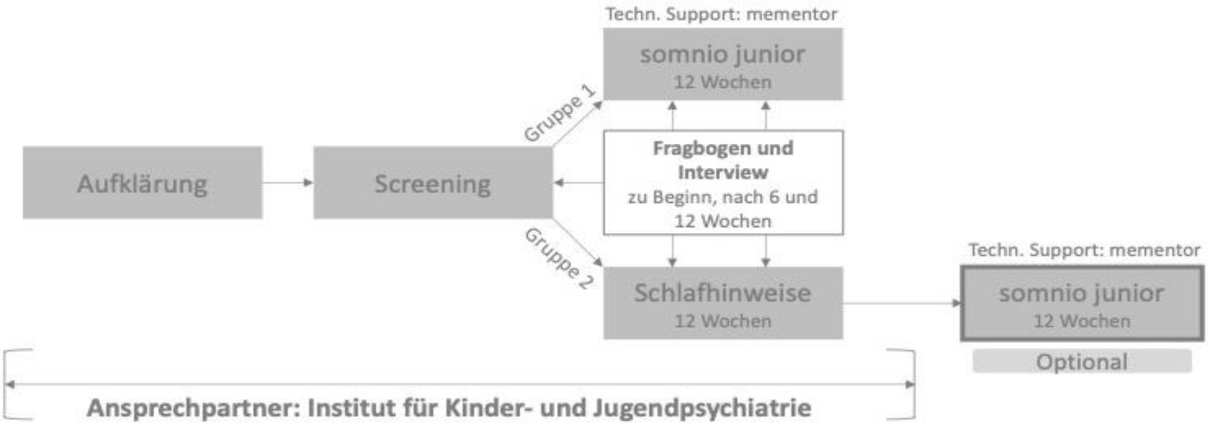

Abbildung 2: Ablauf der Prüfung

Beim ersten online-Termin findet ein **Aufklärungsgespräch** statt, in welchem wir Ihrem Kind und Ihnen die Prüfung vorstellen. Es ist wichtig, dass nicht nur Ihr Kind, sondern auch Sie als Sorgeberechtigte an dem Termin teilnehmen. Vor der Teilnahme an der Prüfung müssen sowohl Ihr Kind als auch Sie als Sorgeberechtigte schriftlich zustimmen. Das online-Aufklärungsgespräch findet auf einem gesicherten Server der Christian-Albrecht-Universität zu Kiel (CAU) statt, für dieses Gespräch erhalten Sie im Vorfeld eine Einladung per E-Mail zu dem online-Besprechungsraum. Von unserer Seite nimmt an dem Gespräch eine Prüfärztin teil. In diesem Gespräch haben wir Zeit für Fragen und um zu klären, ob Ihr Kind Lust hast, an der Prüfung teilzunehmen. Falls Sie alle sich für die Teilnahme an der Prüfung entscheiden, erhalten Sie und Ihr Kind im Anschluss an das Aufklärungsgespräch die **Einwilligungserklärung** für die Teilnahme an der Prüfung per E-Mail zum digitalen Unterschreiben zugeschickt. Wenn Sie die Einwilligung unterschrieben haben, folgt einige Tage später der zweite online-Termin. Beim zweiten online-Termin treffen wir uns wieder im online-Besprechungsraum und es findet ein **Screening** statt Für Jugendliche unter 16 Jahren gilt, dass mindestens ein Sorgeberechtigter bei diesem Gespräch anwesend sein muss. Jugendliche über 16 Jahren können über die Anwesenheit eines Sorgeberechtigten frei entscheiden. Von unserer Seite nehmen an dem Termin eine Psychologin und eine Prüfärztin teil. Bei diesem Termin stellen wir fest, ob Ihr Kind an der Prüfung teilnehmen kann, denn wir haben wissenschaftliche Vorgaben, wer an der Prüfung teilnehmen kann und wer nicht. Wir werden einige allgemeine Fragen sowie speziell Fragen zum Schlafverhalten und zur psychischen Gesundheit stellen. Im Anschluss an das Gespräch werden wir den Link zu einem Fragebogen verschicken, wir bitten Ihr Kind, diesen anschließend online auszufüllen. Das Gespräch und die Beantwortung des online-Fragebogens werden insgesamt ca. 1 Stunde dauern. Die Angaben helfen uns dabei zu entscheiden, ob die Voraussetzungen zur Teilnahme erfüllt sind. Diese und auch sonst alle anderen Angaben sind streng vertraulich und werden nach dem Datenschutzgesetz behandelt. Wenn

wir festgestellt haben, dass eine Teilnahme an der Prüfung möglich ist, wird Ihr Kind zufällig einer von zwei Gruppen zugeordnet. Dieses Vorgehen nennt man Randomisierung.“ Die Gruppe 1 erhält sofort für 12 Wochen den vollen Zugang zur App „somnio junior“. Die Gruppe 2 erhält zunächst für 12 Wochen einfache, aber wertvolle Hinweise zum Schlafverhalten und erst im Anschluss an die Prüfung (nach 12 Wochen) den Zugang zu „somnio junior“. Wir haben uns für diese zwei Gruppe entschieden, damit wir im direkten Vergleich der beiden Gruppen besser verstehen können, was innerhalb von 12 Wochen besser geholfen hat: „somnio junior“ oder die Hinweise zum Schlafverhalten. Das bedeutet aber auch, dass unabhängig von der Gruppenzuteilung alle Teilnehmer Zugang zur App „somnio junior“ erhalten werden.

Gruppe 1: Kurz nach dem Screening erhalten die Teilnehmer von uns per E-Mail einen Zugangscode zur App „somnio junior“ zugeschickt. Jetzt kann die App auf das Smartphone, Tablet oder auf den Computer geladen und den Anweisungen zur Anmeldung gefolgt werden. Hierzu gehört auch, dass den Allgemeinen Geschäftsbedingungen (AGB) der App somnio junior zugestimmt werden muss. Den AGB müssen speziell Sie als Sorgeberechtigte zustimmen. Die AGB finden Sie weiter unten in diesem Dokument. Die App selbst beinhaltet mehrere Module, die selbstständig zu Hause am Smartphone, Tablet oder Computer bearbeitet werden können. Die App nutzt die Ziele und Einträge der Jugendlichen, die im sogenannten Morgen- und Abendprotokoll dokumentiert werden. Wir werden mithilfe der App über den gesamten Zeitraum von 12 Wochen die Nutzung und damit das Schlafverhalten beobachten. Falls es Schwierigkeiten bei der Nutzung der App gibt, kann jederzeit mit dem Support der Firma mementor oder dem Team der Prüfung Kontakt aufgenommen werden - alle Kontaktdaten sind weiter unten aufgelistet. Falls es in der Zeit der Nutzung der App zu gesundheitlichen Problemen irgendwelcher Art kommen sollte, melden Sie oder Ihr Kind diese bitte direkt an uns. Diese gesundheitlichen Probleme können direkt mit dem Schlaf zusammenhängen oder auch nur ein einfacher Schnupfen sein, bitte berichten Sie uns alle gesundheitlichen Probleme. Wir werden sechs Wochen nach Beginn der Prüfung Kontakt mit den Jugendlichen aufnehmen, um Fragen zu dem Verlauf der letzten Wochen und der App zu stellen (Zwischeninterview), anschließend werden wir den Link zu einem online-Fragebogen versenden. Wenn danach weiterhin alles problemlos läuft, werden wir uns selbst erst nach Ablauf der 12 Wochen wieder melden, um das Abschlussinterview durchzuführen und ein letztes Mal den Fragebogen ausfüllen zu lassen.

Gruppe 2: Alle Jugendlichen der Gruppe 2 erhalten per E-Mail eine PDF-Broschüre mit Tipps zum Thema Schlaf. Nach sechs und 12 Wochen melden wir uns bei den Jugendlichen für die Zwischen- bzw. Abschlusserhebung und versenden jeweils einen online-Fragbogen, den wir die Teilnehmenden bitten, auszufüllen. Falls es in der Zeit der Anwendung der Schlafhinweise zu gesundheitlichen Problemen irgendwelcher Art kommen sollte, melden Sie oder Ihr Kind diese bitte direkt an uns. Diese

gesundheitlichen Probleme können direkt mit dem Schlaf zusammenhängen oder auch nur ein einfacher Schnupfen sein, bitte berichten Sie uns alle gesundheitlichen Probleme. Die Jugendlichen in der Gruppe zwei erhalten im Anschluss an die Prüfung (nach 12 Wochen bzw. im Anschluss an die Abschlusserhebung) den Zugangscode zur App „somnio junior". Die App ist dann auch für die Gruppe 2 freigeschaltet und kann frei für 12 Wochen außerhalb der klinischen Prüfung genutzt werden.

Welchen Nutzen hat Ihr Kind von der Teilnahme an der klinischen Prüfung?

Wir hoffen natürlich sehr, dass die App oder auch die Hinweise zum Schlafverhalten helfen, den Schlaf Ihres Kindes zu verbessern. Jedoch können wir dies nicht mit Sicherheit versprechen. In jedem Fall helfen Sie und Ihr Kind uns damit zu verstehen, ob die App den Schlaf verbessern kann.

Welche Risiken sind mit der Teilnahme an der klinischen Prüfung verbunden?

Im Zusammenhang mit der Prüfung kann die intensivere Beschäftigung mit dem Schlaf dazu führen, dass sich die Müdigkeit zeitweilig verstärkt. Einige der Übungen könnten Einfluss auf den Tagesablauf haben, sodass sich Ihr Kind zeitweise vielleicht müder fühlt. Dies wiederum könnte die Stimmung und Leistungsfähigkeit in Schule, Ausbildung oder Beruf beeinflussen. Es könnte aufgrund vermehrter Müdigkeit auch zu Unfällen kommen. Wir bitten Sie daher, diese unerwünschten Veränderungen gut zu beobachten und dem Studienteam immer umgehend mitzuteilen. Natürlich können Sie oder Ihr Kind jederzeit die Entscheidung treffen, nicht weiter an der Prüfung teilzunehmen. Für die Nutzung der App „somnio junior" ist eine Internetverbindung über WLAN oder mobile Daten erforderlich. Es ist jedoch nicht notwendig, dass das Gerät dauerhaft mit dem Internet verbunden ist, sondern nur während der Nutzung der App. Eventuell anfallende Roaming-Gebühren während eines Auslandsaufenthalts müssen selbst getragen werden. Bitte prüfen Sie Ihren Mobilfunktarif, um unerwartete Kosten zu vermeiden.

Wer kann an der klinischen Prüfung teilnehmen?

- Personen, die unter Ein- oder Durchschlafproblemen leiden
- Personen, die zwischen 14 und 17 Jahren alt sind
- Personen mit Zugang zu einem Tablet/Smartphone mit einem Internetzugang
- Personen, welche die deutsche Sprache sprechen und lesen können

Was sind Ausschlusskriterien für die Teilnahme an der klinischen Prüfung?

- Akute Suizidalität

- Epilepsie
- Bipolare Störung
- körperliche Erkrankungen, die einen maßgeblichen Einfluss auf den Schlaf haben können z.B. infolge eines vermehrten Juckreizes (bei akuten oder chronischen Hauterkrankungen wie Neurodermitis, Läusebefall und Krätze) sowie Schmerz- oder Atemwegserkrankungen
- Vollstationäre psychiatrische Krisenintervention innerhalb der letzten vier Wochen
- Suchterkrankungen inklusive Medienbezogene Störung
- Teilnahme an einer anderen klinischen Prüfung

Schwangerschaft

Wenn während der Studie eine Schwangerschaft eintritt, informieren Sie bitte umgehend das Studienzentrum. In diesem Fall muss die Teilnahme an der Studie beendet werden.

Gibt es eine Aufwandsentschädigung für die Teilnahme an der klinischen Prüfung

Ja, Ihr Kind erhält für jeden der drei Terminblöcke Screening, Zwischenerhebung (Interview und Fragebogen) und Abschlusserhebung (Interview und Fragebogen) 10 Euro in Form eines Wertgutscheins. Ihr Kind erhält, nach der Teilnahme an allen drei Terminen so insgesamt einen Wertgutschein in Höhe von 30 Euro. Falls Ihr Kind nicht an allen Terminblöcken teilgenommen hat, wird der Wertgutschein anteilig ausgegeben. Die einmalige Ausgabe des Wertgutscheins findet im Anschluss an die Prüfung statt.

Freiwilligkeit:

Die Teilnahme Ihres Kindes an dieser Prüfung ist freiwillig. Es wird also nur dann in die Prüfung eingeschlossen, wenn Sie und Ihr Kind dazu ihre Einwilligung gegeben haben. Sie können Ihre Einwilligung zur Teilnahme an der Prüfung jederzeit ohne Angaben von Gründen zurücknehmen. Wir werden Sie allerdings einmalig per Telefon/E-Mail kontaktieren, um nach dem Grund für Ihre Beendigung der Prüfung zu fragen. Sie müssen uns darauf keine Antwort geben. Sofern Ihr Kind nicht an der Prüfung teilnehmen oder später aus ihr ausscheiden möchte, entstehen hieraus keine Nachteile.

Datenschutz:

Wenn Sie und Ihr Kind sich entschlossen haben, an der Prüfung teilzunehmen, möchten wir Sie bitten, eine Einwilligungserklärung zu unterschreiben. Der Datenschutz innerhalb der Prüfung ist durch das Datenschutzgesetz (die Europäische Datenschutz-Grundverordnung) genau geregelt. Weiter unten finden Sie noch genauere Informationen zum Datenschutz. Da zur Teilnahme an der Prüfung die

Nutzung der App „somnio junior“ notwendig ist, sind die Allgemeinen Geschäftsbedingungen (AGB) der App weiter unten beigefügt. Die Einwilligung in die AGB der App finden Sie ebenfalls weiter unten.

Informationen zum Datenschutz

Was geschieht mit den Daten, die über die Teilnehmer erhoben werden?

Allgemeine Informationen: Während der klinischen Prüfung werden medizinische Befunde (Gesundheitsdaten etc.) sowie persönliche Daten (Alter, Geschlecht) von den Teilnehmern in der Prüfstelle gespeichert. Alle Mitarbeitenden unterliegen der Schweigepflicht und der Einhaltung des Datenschutzes. Die für die klinische Prüfung wichtigen Daten werden zusätzlich in pseudonymisierter Form gespeichert. Das bedeutet, dass Daten der Teilnehmer nicht unter dem richtigen Namen, sondern unter einem individuellen Studiencode gespeichert werden. Die Referenzliste wird in abschließbaren Datenschränken des Instituts für Kinder- und Jugendpsychiatrie gesichert gelagert. Damit können die Daten nicht mehr dem Namen zugeordnet werden, wodurch die Daten ab diesem Moment anonymisiert sind. Eine Veröffentlichung der Daten erfolgt nur in anonymisierter Form, sodass der Datenschutz in voller Weise gewährleistet ist. Die anonymisierten Daten werden auf einem geschützten Datenträger gespeichert und nach zehn Jahren gelöscht. Im Zusammenhang mit der Nutzung der App „somnio junior“ werden Nutzungsdaten entstehen, die mit in unsere Analyse eingeschlossen werden. Die Firma mementor DE GmbH wird die generierten Daten am Ende der Erhebung an das Studienteam übermitteln.

Rechtsgrundlage: Rechtsgrundlage für die Datenverarbeitung ist Ihre informierte Einwilligung gemäß Art. 6 Abs. 1 Buchst. a und Art. 9 Abs. 2 Buchst. a der EU Datenschutzgrundverordnung (DSGVO) sowie § 29 Medizinprodukterecht-Durchführungsgesetz (MPDG). Die Bereitstellung Ihrer personenbezogenen Daten ist freiwillig. Ohne Ihre ausdrückliche Einwilligung in die Verarbeitung Ihrer Daten können Sie allerdings nicht an dieser klinischen Prüfung teilnehmen.

Verantwortlichkeit: Verantwortlich im Sinne des Datenschutzrechts ist der Sponsor (mementor DE GmbH) ebenso wie die Prüfstelle (Institut für Kinder- und Jugendpsychiatrie) und die Prüfärztin (Telke Schoone).

Zweck: Mit Hilfe der erhobenen Daten soll die Unbedenklichkeit oder Wirksamkeit der App „somnio junior“ bei Jugendlichen zwischen 14-17 Jahren mit einer Insomnie klinische untersucht werden.

Weitergabe/Empfänger: Die für die klinische Prüfung wichtigen Daten werden in pseudonymisierter Form verarbeitet und gegebenenfalls weitergegeben.

Die erhobenen Daten werden, soweit erforderlich, pseudonymisiert weitergeben an:

1. den Sponsor (mementor DE GmbH, Karl-Heine-Straße 15 04229 Leipzig) und von diesem beauftragten Stellen zum Zweck der Durchführung und wissenschaftlichen Auswertung
2. an den Hersteller (mementor DE GmbH, Karl-Heine-Straße 15 04229 Leipzig), wenn die Prüfungsergebnisse verwendet werden sollen, um zu bewerten, ob das Produkt die Kriterien erfüllt, um in das Verzeichnis für digitale Gesundheitsanwendung aufgenommen zu werden.
3. im Falle unerwünschter Ereignisse: an den Sponsor, die zuständige Ethik-Kommission, andere Prüfer
4. im Fall eines schwerwiegenden unerwünschten Ereignisses, das einen Kausalzusammenhang mit dem Prüfprodukt, einem Vergleichsprodukt oder dem Prüfverfahren aufweist, oder bei dem ein Kausalzusammenhang durchaus möglich ist, nach Artikel 80 Absatz 2 der Verordnung (EU) 2017/745 vom Sponsor über das elektronische System nach Artikel 73 der Verordnung (EU) 2017/745 an die Behörden anderer Mitgliedstaaten der Europäischen Union,
5. an die für die Zulassung, Bewertung oder Überwachung zuständige Behörde

Die im Rahmen der oben genannten klinischen Prüfung erhobenen und gespeicherten Daten (auch die originalen Klardaten) können, soweit erforderlich und gesetzlich erlaubt, durch die zuständige Überwachungsbehörde im Rahmen von Inspektionen oder Beauftragte des Sponsors (sog. Auditoren oder Monitore) zur Überprüfung der ordnungsgemäßen Durchführung der klinischen Prüfung in der Prüfstelle eingesehen werden. Diese sind zur Vertraulichkeit verpflichtet, eine Weitergabe der erhobenen Daten erfolgt in diesem Zusammenhang nicht.

Ihre Rechte: Sie haben grundsätzlich folgende Rechte bezüglich Ihrer personenbezogenen Daten, sofern dies nicht, aufgrund einer zwischenzeitlich vorgenommenen Löschung der identifizierenden Merkmale zur Entschlüsselung, technisch oder anderweitig gesetzlich unmöglich ist.

Recht auf Widerruf Ihrer Einwilligung: So wie die Einwilligung zur Teilnahme an der klinischen Prüfung können Sie auch Ihre Einwilligung zur Verarbeitung der erhobenen Daten jederzeit widerrufen. Gemäß § 29 Nr. 2 MPDG dürfen im Falle eines Widerrufs die gespeicherten Daten Ihres Kindes jedoch weiterverwendet werden, soweit dies erforderlich ist, um

1. die Wirkungen des zu prüfenden Arzneimittels festzustellen,

2. sicherzustellen, dass Ihre schutzwürdigen Interessen nicht beeinträchtigt werden,

3. der Pflicht zur Vorlage vollständiger Zulassungsunterlagen zu genügen.

Im Falle eines Widerrufs Ihrer Einwilligung werden die verantwortlichen Stellen unverzüglich prüfen, inwieweit die gespeicherten Daten noch erforderlich sind. Nicht mehr benötige Daten werden unverzüglich gelöscht, sofern nicht gesetzliche und/oder behördliche Dokumentations- und Meldepflichten entgegenstehen. Die bis zum Widerruf erfolgte Datenverarbeitung bleibt jedoch rechtmäßig.

Sie haben weiterhin folgende Rechte: Recht auf Auskunft (inkl. unentgeltlicher Überlassung einer Kopie) über diepersonenbezogenen Daten Ihres Kindes, die im Rahmen der klinischen Prüfung erhoben, verarbeitet oder ggf. an Dritte übermittelt werden. Recht auf Datenübertragung der zur Person Ihres Kindes erhobenen Daten an eine bestimmte Stelle. Recht auf Berichtigung unrichtiger personenbezogener Daten, auf Einschränkung der Verarbeitung und auf Widerspruch gegen die Nutzung der Daten.

Mögliche Einschränkungen Ihrer Rechte: Da die Daten im Rahmen einer klinischen Prüfung mit einem Medizinprodukt gemäß den Bestimmungen des Medizinprodukterecht-Durchführungsgesetzes verwendet werden, können die oben genannten Rechte unter Umständen nach Prüfung des Einzelfalls eingeschränkt werden (insbesondere nach Art. 17 Abs. 3 Buchst. d und Art. 89 DSGVO). Dies gilt insbesondere, wenn der Anwendung eines dieser Rechte vertragliche, gesetzliche und/oder behördliche Dokumentations- und Meldepflichten entgegenstehen oder die Durchführung der klinischen Prüfung hierdurch unmöglich gemacht oder ernsthaft beeinträchtigt würde.

Wahrnehmung Ihrer Rechte: Wollen Sie von einem oder mehreren der genannten Rechte Gebrauch machen, kontaktieren Sie bitte direkt das Studienteam (Tel.: 0431 500 -98345, Mail: studie.somnio.kiel@uksh.de). Bei Anliegen zur Datenverarbeitung und zur Einhaltung der datenschutzrechtlichen Anforderungen können Sie sich auch an die Datenschutzbeauftragten des Prüfzentrums oder Sponsors wenden (siehe Kontaktdaten, Seite 14).

Sie haben grundsätzlich auch das Recht, jederzeit den Sponsor selbst zu kontaktieren. Bitte wenden Sie sich jedoch im Regelfall an den Prüfer bzw. den Datenschutzbeauftragten Ihres Prüfzentrums, da aufgrund der Pseudonymisierung nur hier Ihre Identität bekannt ist und damit sinnvollerweise weitere Schritte unternommen werden können, bzw. eine unbeabsichtigte Identifikation der Person Ihres Kindes durch den Sponsor vermieden werden kann. Sie haben außerdem ein Beschwerderecht bei einer

Datenschutzaufsichtsbehörde. Sollten Sie Bedenken hinsichtlich des Umgangs mit den personenbezogenen Daten Ihres Kindes haben, können Sie sich an folgende Stellen wenden:
Datenschutzaufsichtsbehörde des Bundeslandes, in dem Ihr Prüfzentrum liegt:
Unabhängiges Landeszentrum für Datenschutz Schleswig-Holstein, Postfach 7116, 24171 Kiel.
Für den Sponsor bzw. dessen Vertreter innerhalb der EU zuständige Datenschutzaufsichtsbehörde: Sächsische Datenschutz- und Transparenzbeauftragte, Postfach 11 01 32, 01330 Dresden.

Dauer der Speicherung der Daten: Die erhobenen Daten sind vom Sponsor auf Grund der Vorgaben in Anhang XV Kapitel III Abschnitt 3 der Verordnung (EU) 2017/745 über einen Zeitraum von mindestens zehn Jahren nach Beendigung der klinischen Prüfung mit dem betreffenden Produkt oder — falls das Produkt anschließend in Verkehr gebracht wird — mindestens zehn Jahren nach dem Inverkehrbringen des letzten Produkts aufzubewahren. Der Sponsor und die Prüfstelle speichern die personenbeziehbaren Daten Ihres Kindes entsprechend mindestens zehn Jahre. Danach werden die personenbezogenen Daten Ihre Kindes gelöscht.

Veröffentlichung**:** Wissenschaftliche Veröffentlichungen von Ergebnissen erfolgen in einer Form, die keine direkten Rückschlüsse auf Personen zulässt.

Allgemeine Geschäftsbedingungen der App somnio junior

1	**Allgemeines**
•	Du schließt eine Nutzungsvereinbarung mit der mementor DE GmbH (nachfolgend mementor). Indem Du diese AGB akzeptierst, werden diese Vertragsbestandteil in Bezug auf die Nutzung des Online-Programms somnio junior.
2	**Gegenstand**
•	Gegenstand dieser AGB ist die Nutzung des Online-Programms *somnio junior*. *somnio junior* bietet Dir Informationen und Übungen rund um das Thema Schlafstörungen, die auf wissenschaftlich geprüften, psychotherapeutischen Methoden basieren.
•	Wir weisen ausdrücklich darauf hin, dass die Nutzung von *somnio junior* kein Ersatz für eine Diagnose oder eine ärztliche bzw. psychotherapeutische Behandlung ist.
3	**Zugangsberechtigung**
•	Die Voraussetzung für die Nutzung von *somnio junior* ist eine Registrierung. Mit der Registrierung werden die AGB und die Datenschutzerklärung anerkannt. Wenn Du jünger als 18 Jahre alt bist, muss mindestens einer Deiner gesetzlichen Vertreter (in der Regel sind das Deine Eltern) schriftlich eingewilligt haben. mementor kann Dich jederzeit auffordern, einen Nachweis über Deine Identität, Dein Alter und/oder die Zustimmung Deiner gesetzlichen Vertreter vorzulegen. Die AGB treten in Kraft, sobald die Registrierung für *somnio junior* abgeschlossen wurde.
•	Die Registrierung ist nur mit einem Lizenzcode möglich.
4	**Deine Rechte**
•	Für die Nutzung von *somnio junior* erhältst Du das einfache, nicht ausschließliche und auf Dritte nicht übertragbare Nutzungsrecht. Ein Erwerb an den Inhalten ist damit nicht verbunden.
•	*somnio junior* steht Dir während der Lizenzlaufzeit grundsätzlich durchgehend zur Verfügung. Aus technischen Gründen (z. B. wegen routinemäßiger oder erforderlicher Wartungsarbeiten) kann die Verfügbarkeit von *somnio junior* zeitweise eingeschränkt sein.
5	**Deine Pflichten**
•	Für den Zugang zum Programm benötigst Du individuelle Anmeldedaten, für deren Geheimhaltung Du verantwortlich bist und deren Missbrauch Du zu verhindern hast. Bei Missbrauch jeglicher Art hast Du mementor hierüber zu informieren über die E-Mail-Adresse support@junior-somni.io. mementor ist bei einer Vertragsverletzung dazu berechtigt, den betroffenen Zugang zu sperren. Du haftest für einen von Dir zu vertretenden Missbrauch.
•	Die für die Nutzung von *somnio junior* anfallenden Endgerätekosten (z.B. Laptop) und Telekommunikationsentgelte (z.B. Kosten für den Internetzugang) sind von Dir zu tragen. Für eine Nutzung außerhalb dieses Landes können entsprechende Roaming-Gebühren anfallen, die von Dir zu tragen sind.
•	Erforderliche Software (z.B. Betriebssystem oder Internetbrowser), die für die Nutzung von *somnio junior* notwendig ist, ist von Dir zu installieren. Des Weiteren bist Du dafür verantwortlich, die technischen Voraussetzungen für den Zugang zu *somnio junior* zu schaffen, insbesondere hinsichtlich der eingesetzten Hardware, der Betriebssystemsoftware, der Verbindung zum Internet und der Browsersoftware.
•	Im Falle der Weiterentwicklung von *somnio junior* bist Du dafür verantwortlich, die erforderlichen Anpassungen bei der von Dir eingesetzten Hard- und Software zu treffen (z.B. Aktualisierung des Browsers).
•	Solltest Du Dich aus irgendeinem Grund tagsüber sehr müde fühlen, solltest Du auf das Autofahren und auf das Bedienen von Maschinen verzichten.
6	**Vertragsverletzungen**
•	Im Falle einer Vertragsverletzung Deinerseits ist mementor zur fristlosen Kündigung berechtigt. Schadensersatzansprüche bleiben hiervon unberührt
7	**Haftungsausschluss**
•	mementor haftet bei Vorsatz und grober Fahrlässigkeit nach den gesetzlichen Bestimmungen. Bei leicht fahrlässig verursachten Verletzungen wesentlicher Vertragspflichten haftet mementor für den vorhersehbaren, vertragstypischen Schaden. Wesentliche Vertragspflichten sind solche, die für die ordnungsgemäße

Durchführung des Vertragszweckes von besonderer Bedeutung sind und auf deren Einhaltung der Kunde vertrauen durfte. Im Übrigen ist die Haftung für leichte Fahrlässigkeit ausgeschlossen

- Für von mementor nicht verschuldete Störungen innerhalb des Leitungsnetzes übernimmt Firma keine Haftung.
- Die Haftung aufgrund zwingender gesetzlicher Vorschriften, einschließlich derjenigen des Produkthaftungsgesetzes, bleibt unberührt.
- Du bist für eine regelmäßige Sicherung Deiner Daten verantwortlich. Bei einem Datenverlust haftet mementor höchstens in Höhe des Aufwands, der zur Wiederherstellung der Sicherungen von deinen Daten notwendig ist.
- Die mementor-Apps bzw. unsere Webseite können auch Links oder Verweise auf Internet-Seiten Dritter enthalten, auf deren Inhalt wir keinen Einfluss haben. Deshalb übernehmen wir trotz sorgfältiger inhaltlicher Kontrolle keine Haftung für die Inhalte externer Links. Für den Inhalt der verlinkten Seiten sind ausschließlich deren Betreiber verantwortlich.
- Die individuellen Unterschiede unter allen Nutzer:innen und in jeder körperlichen Verfassung sind vielfältig. Die Gesundheit eines jedes Einzelnen hängt von einer Vielzahl verschiedener Faktoren ab. Auch der Erfolg eines durch mementor zur Verfügung gestellten digitalen Programms ist von verschiedenen Faktoren abhängig, auf die mementor keinen Einfluss hat. Folglich kann keine Garantie für den Erfolg eines App-Programms übernommen werden.
- Eine Haftung von mementor für die Richtigkeit des digitalen Programms ist ausgeschlossen, sofern Du falsche oder nicht vollständige Angaben, insbesondere zum Gesundheitszustand, zu Vorerkrankungen oder dem Vorliegen von Erkrankungen jeglicher Art gemacht hast oder es unterlassen hast, solche Angaben zu machen.
- Eine Haftung von mementor ist ausgeschlossen, sofern die Inhalte des Programms entgegen ihrer Zweckbestimmung zu einer Selbstdiagnose und/oder -behandlung herangezogen werden. Das Gleiche gilt, falls aufgrund der erteilten Informationen und Auskünfte die notwendige Konsultation einer Fachperson nicht stattfindet.

8 Urheberrecht

- Die Inhalte des Online-Programms *somnio junior* unterliegen dem Urheberrecht von mementor. Die Inhalte beruhen auf wissenschaftlich anerkannten psychologischen Methoden, die von psychologischen Expert:innen entwickelt worden sind.
- Du hast die Möglichkeit, Material und Informationen für den persönlichen Gebrauch herunterzuladen. Dieses Material ist ausschließlich zu Deiner eigenen Nutzung bestimmt und darf nicht an Dritte weitergegeben oder kommerziell verbreitet werden. Jede Verbreitung, Veränderung oder Reproduktion von Material und Informationen ist nicht gestattet.
- Bilder, Graphiken und das Layout von *somnio junior* sind Eigentum von mementor und dürfen ohne vorherige schriftliche Genehmigung nicht reproduziert oder verbreitet werden.

9 Aktualisierungen/Änderungen

- mementor behält sich vor, ohne vorherige Ankündigung Änderungen oder Ergänzungen der bereitgestellten Informationen vorzunehmen. Eine Haftung ergibt sich daraus nicht.

10 Schlussbestimmungen

- Es gilt deutsches Recht.
- Gerichtsstand für alle Streitigkeiten aus diesem Vertragsverhältnis ist Leipzig.
- Sollten einzelne Regelungen dieser AGB unwirksam oder anfechtbar sein, wird dadurch die Wirksamkeit der übrigen Regelungen nicht berührt.

11 Kontakt

- Du kannst uns über Email oder telefonisch kontaktieren: support@junior-somnio.io / 0341 97852812.

Wem können Sie weitere Fragen stellen?

Wenn Sie Fragen haben, dann melden Sie sich bitte einfach beim Studienteams (Tel.: 0431 500 98345, Mail: studie.somnio.kiel@uksh.de). Sie beantworten gerne Ihre Fragen im Zusammenhang mit der Prüfung. Bei technischen Fragen im Zusammenhang mit der Nutzung der App melden Sie sich gern beim Support-Team (Tel.: 0341 97852812, Mail: support@junior-somnio.io).

Kontaktdaten
**Studienverantwortliche**<br>Prof. Dr. Alexander Prehn-Kristensen<br>Stellvertretender Direktor<br>Institut für Kinder- und Jugendpsychiatrie<br>Zentrum für Integrative Psychiatrie ZIP gGmbH, Campus Kiel<br>Universitätsklinikum Schleswig-Holstein<br>Niemannsweg 147, 24105 Kiel<br>Tel.: 0431 500 98316<br>Mail: alexander.prehn-kristensen@uksh.de<br><br>M. Sc. Psych. Beke Ralfs<br>Wissenschaftliche Mitarbeiterin<br>Institut für Kinder- und Jugendpsychiatrie<br>Zentrum für Integrative Psychiatrie ZIP gGmbH, Campus Kiel<br>Universitätsklinikum Schleswig-Holstein<br>Niemannsweg 147, 24105 Kiel<br>Tel.: 0431 500 98345<br>Mail: beke.ralfs@uksh.de
**mementor DE GmbH**<br>Kontaktdaten des Datenschutzbeauftragten<br>Ansprechpartner: Paul Schmude<br>Karl-Heine-Straße 15, 04229 Leipzig<br>dataprivacy@mementor.de<br><br>**Technischer Support**<br>Kontaktdaten vom Technischen Support<br>Tel.: 0341 97852812<br>Mail: support@junior-somnio.io
**Prüfärztin**<br>Telke Schoone<br>Klinik für Psychiatrie, Psychotherapie und Psychosomatik des Kindes- und Jugendalters<br>Zentrum für Integrative Psychiatrie ZIP gGmbH<br>Universitätsklinikum Schleswig-Holstein, Campus Kiel<br>Niemannsweg 147, 24105 Kiel<br>Telefon: 0431 500 98344<br>Mail: telke.Schoone@uksh.de
**Empfänger der personenbezogenen Daten Ihres Kindes**<br>Die Daten werden von uns (Institut für Kinder- und Jugendpsychiatrie am ZIP) verarbeitet.
**Datenschutzbeauftragte der Christian-Albrechts-Universität zu Kiel (CAU)**<br>Frau Stella Thoben<br>Tel.: 0431 880 3581<br>Mail: sthoben@uv.uni-kiel.de
**Landesbehörde Schleswig-Holstein**<br>Unabhängiges Landeszentrum für Datenschatz (ULD) Schleswig-Holstein<br>Ansprechpartnerin: Marit Hansen, Landesbeauftragte für Datenschutz Schleswig-Holstein<br>Holstenstraße 98, 24103 Kiel<br>Tel.: 0431 988 1200<br>Mail: mail@datenschutzzentrum.de

Versicherungsschutz

Bei der klinischen Prüfung eines Medizinproduktes müssen alle Studienteilnehmer gemäß dem Medizinproduktegesetz versichert sein. Der Umfang des Versicherungsschutzes ergibt sich aus den Versicherungsunterlagen, die Sie ausgehändigt bekommen. Wenn Sie vermuten, dass durch die Teilnahme an der klinischen Prüfung Ihre Gesundheit oder die Ihres Kindes geschädigt oder vorher bestehende Leiden verstärkt wurden, müssen Sie dies unverzüglich dem Versicherer

Name und Anschrift der Versicherung:

Name: HDI Global SE

Telefon: 0211 7482 246

Fax: 0511 645 1150197

Versicherungsnummer: 76601168 03202

direkt anzeigen, gegebenenfalls mit Unterstützung durch Ihren Prüfarzt, um den Versicherungsschutz nicht zu gefährden. Sofern der Prüfarzt oder die Prüfärztin Sie dabei unterstützt, erhalten Sie eine Kopie der Meldung. Sofern Sie die Anzeige direkt an den Versicherer richten, informieren Sie bitte zusätzlich den Prüfarzt oder die Prüfärztin.

Bei der Aufklärung der Ursache oder des Umfangs eines Schadens müssen Sie mitwirken und alles unternehmen, um weiteren Schaden abzuwenden. Während der Dauer der klinischen Prüfung dürfen die Teilnehmer sich einer anderen medizinischen Behandlung – außer in Notfällen – nur nach vorheriger Rücksprache mit dem Prüfarzt oder der Prüfärztin unterziehen. Von einer erfolgten Notfallbehandlung muss der Prüfarzt oder die Prüfärztin unverzüglich unterrichtet werden.

Sie erhalten ein Exemplar der Versicherungsbestätigung einschließlich der Versicherungsbedingungen. Wir weisen Sie insbesondere auf Punkt 1.4 (zu den Ausschlüssen) und Punkt 3 (Leistung des Versicherers) hin. Bitte beachten Sie außerdem die Obliegenheiten unter Punkt 4.3 (das sind Pflichten, die Sie im eigenen Interesse beachten müssen, um Ihren Versicherungsschutz zu erhalten). Wir weisen Sie ferner darauf hin, dass die Teilnehmer auf dem Weg von und zur Prüfstelle nicht unfallversichert sind.

Vielen Dank, dass Sie diese Information bis zum Ende durchgelesen haben.

Wir freuen uns auf Ihre Fragen!

Prüfstelle:

Institut für Kinder- und Jugendpsychiatrie

Zentrum für Integrative Psychiatrie ZIP gGmbH

Universitätsklinikum Schleswig-Holstein, Campus Kiel

Niemannsweg 147, 24105 Kiel

EUDAMED-Nr. CIV-24-05-046919

Eine randomisierte, kontrollierte klinische Prüfung zur Untersuchung der Wirksamkeit der digitalen Kognitiven Verhaltenstherapie *somnio junior* zur Reduzierung der Insomniesymptome bei Jugendlichen mit Insomnie nach dreimonatiger Nutzung

Einwilligungserklärung

____

Vor- und Nachnamen Sorgeberechtigte(r) in Druckbuchstaben

____

Vor- und Nachnamen Sorgeberechtigte(r) in Druckbuchstaben

Ich bin in einem persönlichen Gespräch durch den Prüfarzt/ die Prüfärztin

____

Name der Ärztin/ des Arztes

ausführlich und verständlich über Wesen, Bedeutung, Risiken und Tragweite der klinischen Prüfung aufgeklärt worden. Ich habe darüber hinaus den Text der Patienteninformation mit den Informationen zu gesundheitlichen Aspekten und Informationen zur Verwendung der Daten gelesen und verstanden. Ich hatte die Gelegenheit, mit der Prüfärztin/Prüfarzt über die Durchführung der klinischen Prüfung zu sprechen. Alle meine Fragen wurden zufrieden stellend beantwortet. Ich hatte ausreichend Zeit, mich zu entscheiden. Mir ist bekannt, dass ich jederzeit und ohne Angabe von Gründen meine Einwilligung zur Teilnahme meines Kindes an der Prüfung zurückziehen kann (mündlich oder schriftlich), ohne dass mir oder meinem Kind daraus Nachteile entstehen.

Ergänzungen durch die aufklärende Person:

Folgende wesentliche Gesichtspunkte oder Fragen sind in dem mündlichen Aufklärungsgespräch genauer besprochen worden. Ich habe mich dabei davon überzeugt, dass der Patient und die Sorgeberechtige(n) alle für ihn bzw. sie wichtigen Fragen stellen konnte(n) und die Aufklärung für ihn bzw. sie verständlich war.

Datenschutzrechtliche Einwilligung

Mir ist bekannt, dass bei dieser klinischen Prüfung **personenbezogene Daten,** insbesondere medizinische Befunde, erhoben, gespeichert und ausgewertet werden sollen. Die Verwendung der personenbezogenen Daten setzt vor der Teilnahme an der klinischen Prüfung folgende freiwillig abgegebene Einwilligungserklärung voraus; ohne die nachfolgende Einwilligung kann mein Kind nicht an der klinischen Prüfung teilnehmen.

Ich willige ein, dass im Rahmen dieser klinischen Prüfung personenbezogene Daten meines Kindes, insbesondere Angaben über die Gesundheit, erhoben werden und in Papierform sowie auf elektronischen Datenträgern gemäß der Angaben in der Informationsschrift gespeichert werden.

____

Vor- und Nachnamen Sorgeberechtigte(r) in Druckbuchstaben

____

Vor- und Nachnamen Sorgeberechtigte(r) in Druckbuchstaben

Datenschutzrechtliche Einwilligung: Tonaufzeichnung
Ich willige ein, dass im Rahmen dieser klinischen Studie digitale Tonaufzeichnungen vom Abschlussgespräch meines Kindes mit dem Studienteam gemacht werden. Ich habe verstanden, dass die Aufzeichnung optional ist und mein Kind auch ohne meine Einwilligung in die Tonaufzeichung an der klinischen Studie teilnehmen kann.<br>☐ Ja ☐ nein<br><br>..<br>Datum, Unterschrift Sorgeberechtigte(r)

Einwilligung in die Allgemeine Geschäftsbedingungen der App somnio junior

Ich wurde im Rahmen der Studieninformationen und des Aufklärungsgesprächs über die AGB aufgeklärt. Ein Exemplar der Patienten-Information und -Einwilligung sowie die Versicherungsunterlagen habe ich erhalten. Ein Exemplar verbleibt im Prüfzentrum.

Ich willige freiwillig ein, dass mein Kind an der oben genannten klinischen Prüfung teilnimmt. Zugleich willige ich in die Verarbeitung der personenbezogenen Daten meines Kindes ein und akzeptiere die AGB der App stellvertretend für mein Kind.

____

Vor- und Nachnamen Sorgeberechtigte(r) in Druckbuchstaben

____

Datum, Unterschrift Sorgeberechtigte(r)

____

Vor- und Nachnamen Sorgeberechtigte(r) in Druckbuchstaben

____

Datum, Unterschrift Sorgeberechtigte(r)

Ich habe das Aufklärungsgespräch geführt, die Jugendlichen und ihre Sorgeberechtigten wurden von mir über den Inhalt und Ablauf der Prüfung sowie Vor- und Nachteile der Teilnahme informiert.

____

Name des Prüfarztes/der Prüfärztin in Druckbuchstaben

____

Datum, Unterschrift des Prüfarztes/der Prüfärztin
