# Supplementary material for: Digital treatment for insomnia in adolescents: study protocol for a randomized controlled trial comparing digital cognitive behavioral therapy for insomnia to sleep hygiene
Source: Front Child Adolesc Psychiatry. 2026 May 1;5:1686491. doi: 10.3389/frcha.2026.1686491 (PMC13176156; doi:10.3389/frcha.2026.1686491)
Supplement: Additional File 3 — Consent form for custodians (PDF 518 kb). [file Datasheet3.pdf]

Deine Top 10 Regeln für besseren Schlaf

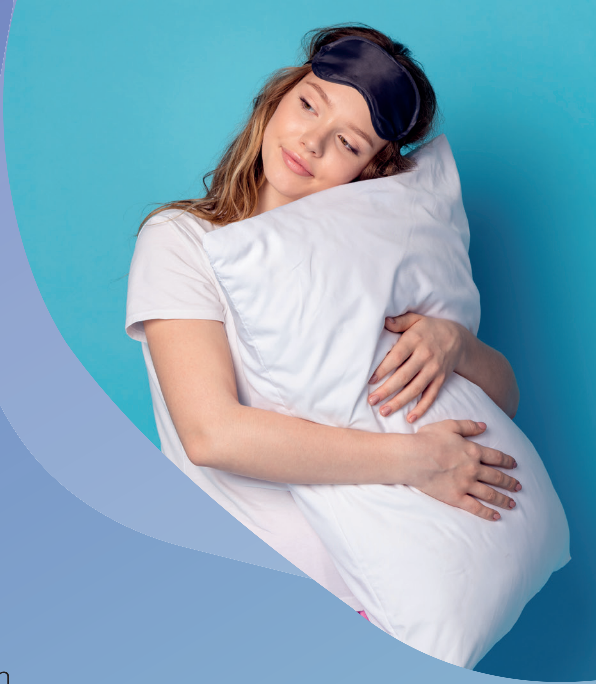

1. Verhalten im Bett

Nutze Dein Bett nur zum Schlafen. Lesen und sexuelle Aktivitäten können eine Ausnahme sein, wenn sie Dein Einschlafen fördern.

2. 15-Minuten-Regel

Wenn Du nach dem Zubettgehen 15 bis 20 Minuten lang nicht einschlafen kannst oder in der Nacht wach liegst, steh auf und geh in einen anderen Raum. Beschäftige Dich mit etwas Ruhigem (z. B. lesen) oder schreibe Deine Gedanken auf. Geh erst zurück ins Bett, wenn etwas Zeit vergangen ist und Du Dich ausreichend schläfrig fühlst.

3. Bett & Beleuchtung

Suche ein für Dich angenehmes Bett. Dabei kannst Du etwas herumexperimentieren (z. B. mit der Anzahl der Kissen). Sorge dafür, dass es wirklich dunkel ist, wenn Du schlafen möchtest.

4. Temperatur

Tatsächlich liegt die ideale Temperatur für Schlaf bei ca. 17 Grad, das Zimmer sollte also nicht zu warm und nicht zu kalt sein.

5. Geräusche

Nächtliche Lärmquellen (z. B. Straßenlärm, laute Uhr) sollten so gut wie möglich beseitigt werden. Dazu kannst Du Ohrstöpsel verwenden, die Lärmquellen beseitigen, oder in ein ruhigeres Zimmer umziehen.

somnio JUNIOR

App bei **Schlafstörungen**

6. Einschlafroutine

Verschiedene Aktivitäten (z. B. Klamotten herauslegen, Hörbuch hören), jeden Abend in der gleichen Reihenfolge durchzuführen kann Dir helfen, zur Ruhe zu finden.

7. Handy

Versuche Deine tägliche Bildschirmzeit auf maximal 2 Stunden zu beschränken.

8. Ernährung

Wenn Du spät am Abend hungrig bist, solltest Du Dir am Besten nur einen leichten Snack genehmigen (z. B. Naturjoghurt mit Haferflocken, Nüssen und etwas Banane), da große und fettige Mahlzeiten sehr schwer im Magen liegen und die Verdauung Deinen Körper am Schlafen hindert.

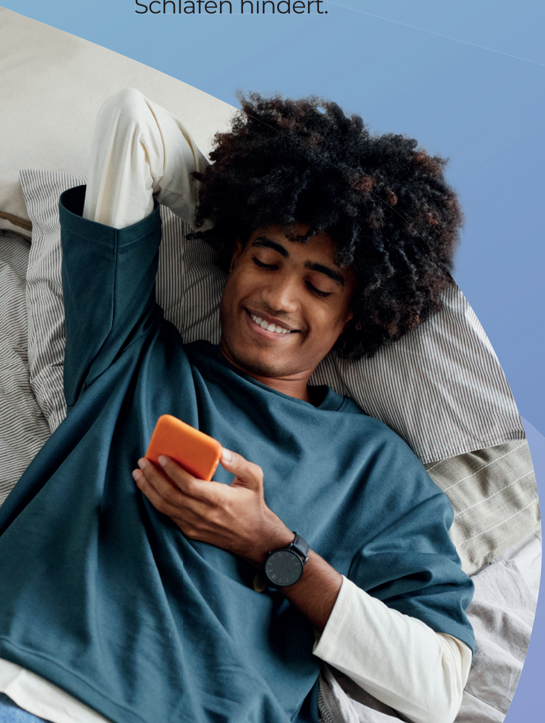

9. Bewegung

Bewegung hilft unserem Schlaf. Bewege Dich idealerweise 60 Minuten bei mäßiger bis intensiver körperlicher Aktivität pro Tag (z. B. schnelles Gehen, Radfahren oder Sport).

10. Substanzen

Vermeide Alkohol, Nikotin, Cannabis und Schlafmittel als Einschlafhilfe. Die Wirkung dieser Substanzen ist trügerisch und sie vermindern langfristig Deine Schlafqualität.

Diese 10 Regeln können Dir dabei helfen, einen guten und erholsamen Schlaf zu bekommen.

Mementor by ResMed
