# Supplementary material for: Digital treatment for insomnia in adolescents: study protocol for a randomized controlled trial comparing digital cognitive behavioral therapy for insomnia to sleep hygiene
Source: Front Child Adolesc Psychiatry. 2026 May 1;5:1686491. doi: 10.3389/frcha.2026.1686491 (PMC13176156; doi:10.3389/frcha.2026.1686491)
Supplement: Additional File 4 — Flyer for the CG “Your Top 10 Rules for better sleep” in German (PDF 1.075 kb). [file Datasheet4.pdf]

(4) Leitfaden und Dokumentation zum Zwischengespräch

**Verantwortliche**:<br>(Prüfer:in/ Study Nurse)	Fragen per Mail an:<br>studie.somnio.kiel@uksh.de

**Screening Nr.**: SOMJU-K01-	**Datum**:

Ziel des Leitfadens

Der Leitfaden zielt darauf ab, dass die telefonischen Interviews mit den Studienteilnehmenden standardisiert und einheitlich ablaufen. So wird sichergestellt, dass alle Studienteilnehmenden gleichbehandelt werden.

Wichtige Hinweise für das Interview

- Es ist wichtig, die Vorgaben des Leitfadens zu befolgen und am Ende des Gesprächs die Befolgung des Leitfadens schriftlich mit Unterschrift zu bestätigen.
- Sollten die Teilnehmenden medizinische/therapeutische Fragen zur Behandlung stellen, sollen die Teilnehmenden darauf hingewiesen werden, dass
    - im Rahmen des Gesprächs keine Therapie/Behandlung stattfinden kann.
    - im Rahmen des Gesprächs vorrangig organisatorische Fragen zur Studie oder allgemeine Verständnisfragen beantwortet werden können.
    - das Gespräch der Erfassung wichtiger Informationen für die Studie dient.
    - sich die Teilnehmenden mit medizinischen Fragen bitte an die Studienärztin oder ihre/n Behandler/Behandlerin wenden sollen.
- Sollten Teilnehmende aus der Interventionsgruppe technische Fragen oder Verständnisfragen zum Produkt haben, können diese, soweit möglich, beantwortet werden. Falls eine Beantwortung nicht möglich ist, können die Teilnehmenden an den mementor Support verwiesen werden.
- Sollten Teilnehmende aus der Kontrollgruppe Verständnisfragen zu ihrer Intervention haben, können diese, sofern möglich, ebenfalls beantwortet werden.
- Es ist bei Rückfragen zur Intervention darauf zu achten, dass die Teilnehmenden nicht beeinflusst (z.B. Motivation zur Nutzung ihrer Intervention) werden

Einleitung

Herzlich willkommen! Das Zwischengespräch wird ungefähr 20 Minuten dauern.

- Ziel des Gesprächs ist es, Informationen für die Studie zu sammeln.
- Im Rahmen des Gesprächs keine Behandlung / Therapie (Wenn konkrete medizinische Fragen vorliegen, sollten diese mit dem/der Behandler:in besprochen werden.)

Ablauf: Ich werde Dir jetzt zunächst einen kurzen Fragebogen freigeben. Danach ein paar Fragen zu dem Befinden in den letzten 6 Wochen stellen.

Dokumentation Erhebung t1

Wir schicken dir jetzt wieder den Link zu deinen Fragebögen zu.

Wichtiger Hinweis: Es dürfen den Teilnehmenden keine Vorgaben, Hinweise oder Anmerkungen zur Beantwortung der Fragen gegeben werden. Während des Ausfüllens darf nicht mit den Teilnehmenden gesprochen werden. Lediglich bei Unklarheiten oder Verständnisfragen seitens der Teilnehmenden darf während des Ausfüllens gesprochen werden.

- Die Teilnehmenden sollen über den Ablauf aufgeklärt werden (Fragebogen per Mail zugeschickt, kurz Bescheid geben sobald Fragebogen angekommen ist) und folgende Hinweise erhalten:
    - Beim Ausfüllen des Fragebogens sollen sich Teilnehmende ganz auf eigene Eindrücke und Erfahrungen stützen. Sollen sich in Ruhe Zeit dafür nehmen.
    - Es gibt keine richtigen oder falschen Antworten.
    - Es ist wichtig, dass der Fragebogen unabhängig und ungestört sowie ohne Beeinflussung von Interviewer:in oder anderen Personen ausgefüllt wird.
    - Während des Ausfüllens wird der/die Interviewer:in nichts proaktiv sagen, aber weiterhin im Online-Meeting bleiben, sollten Rückfragen aufkommen.
- Sobald die Mail mit dem Fragebogen bei den Teilnehmenden angekommen ist, soll der Fragebogen geöffnet werden und der/die Teilnehmende beginnt eigenständig das Ausfüllen der Fragen, sobald er/sie bereit ist, während der/die Interviewer:in nur bei Rückfragen aktiv wird.
- Wenn der Fragebogen vollständig ausgefüllt ist, wird die Nachfrage gestellt, ob der Fragebogen vollständig ausgefüllt und abgeschickt wurde. Falls ja, geht es anschließend weiter mit der Abfrage des Befindens in den letzten 6 Wochen.

☐	Zusendung der Fragebögen für t1 an Teilnehmer:in	Datum:
☐	Fragebögen durch Teilnehmer:in ausgefüllt	Datum:

Anmerkung:
Die Daten aus den Fragebögen der t1-Erhebung liegen nach dem Ausfüllen durch die Teilnehmenden direkt im eCRF vor.

Fragen zu AEs und Veränderungen der Umstände

**Fragen zu deinem Befinden in den letzten sechs Wochen (seit Beginn der Studie)**		
Hattest Du in den letzten 6 Wochen Schulferien?	☐ Nein<br>☐ Ja (Start: Ende:)	
**Frage nach unerwünschten Ereignissen***	Ja	Nein
Sind Dir während der letzten sechs Wochen (seit Beginn der Studie) ungewöhnliche Dinge passiert?	☐	☐
Warst Du in den letzten sechs Wochen (seit Beginn der Studie) krank oder hast Du Dich krank gefühlt?	☐	☐
Falls Ja: Welche Symptome hattest Du? ☐ Kopfschmerzen<br>☐ Schwindel<br>☐ Erkältung<br>☐ Vermehrte Müdigkeit<br>☐ Andere: ____________________________		
Kam es in den letzten sechs Wochen (seit Beginn der Studie) zu Unfällen (z.B. im Straßenverkehr, Zuhause oder in der Schule)	☐	☐
Hast Du Dich in den letzten sechs Wochen (seit Beginn der Studie) vermehrt traurig, ängstlich oder erschöpft gefühlt?	☐	☐
Fragen, um psychische Symptome wie z.B. vermehrte Gereiztheit, Konzentrationsstörungen o.ä. zu erfassen:		
1. Haben sich Deine schulischen Leistungen in den letzten sechs Wochen (seit Beginn der Studie) verschlechtert?	☐	☐
2. Hast Du dich in den letzten sechs Wochen (seit Beginn der Studie) vermehrt mit Deinen Sorgeberechtigten oder Freund:innen gestritten?	☐	☐
Falls Ja bei 1. Oder 2.: Sind diese auf Veränderungen des psychischen Zustandes der Teilnehmenden zurückzuführen?<br>Wenn ja, welche:	☐	☐

* falls es zu einem unerwünschten Ereignis gekommen ist: AE-Bogen ausfüllen

**Beschreibung**<br>(Betroffene Person, Start-/Stoppdatum, Intensität, Schwerwiegend (SAE?), Zusammenhang mit dem Prüfprodukt, Ergriffene Maßnahme, Konsequenz)

**Änderungen Begleitmedikation/-therapie**							Ja	Nein
Gibt es Änderungen in der Begleitmedikation?<br>Falls ja:							☐	☐
Name Med.	Indikation	Dosis [Einheit]	Einnahme-frequenz	Darreichungs-form & Weg der Einnahme	Startdatum	Stoppdatum		
						☐ Andauernd		
						☐ Andauernd		
Anmerkung:								
Gibt es Änderungen in den begleitenden Therapien?<br>Falls ja:							☐	☐
Leitdiagnose:	Insomnie schon Thema?	Setting:	Startdatum:	Stoppdatum:				
	☐ Ja<br>☐Nein	Wählen Sie ein Element aus.		☐ Andauernd				
Anmerkung:								

Maßnahmen zur Verbesserung des Schlafes

	Ja	Nein
Gibt es Änderungen in sonstigen Maßnahmen zur Verbesserung des Schlafs? (z.B. Alkohol, Cannabis, psychologische oder pharmakologische Maßnahmen, die nicht unter Begleitmedikation oder begleitende Therapie fällt)<br>Falls ja, welche:	☐	☐

Abfrage nach Device Deficiencies

**Frage nach Device Deficiencies**	Ja	Nein
Sind Produktmängel (App bzw. Flyer) aufgetreten?	☐	☐

Abschluss des Gesprächs

**Verabschiedung**
Vielen Dank für Deine/Eure Zeit. Wir sind jetzt fertig. Wir werden Sie in ca. 5 Wochen wieder telefonisch kontaktieren, um einen Termin für das Abschlussgespräch zu vereinbaren. Verabschiedung des/der Teilnehmenden.
**Anmerkungen**
**Angabe zum Befolgen des Leitfadens**
☐ Die Vorgaben des Leitfadens wurden befolgt<br>☐ Die Vorgaben des Leitfadens wurden mit Abweichungen befolgt<br>Angabe der Abweichung:<br>Begründung:

Unterschrift: X ______________________

Prüfer:in/ Study Nurse
