# Supplementary material for: Digital treatment for insomnia in adolescents: study protocol for a randomized controlled trial comparing digital cognitive behavioral therapy for insomnia to sleep hygiene
Source: Front Child Adolesc Psychiatry. 2026 May 1;5:1686491. doi: 10.3389/frcha.2026.1686491 (PMC13176156; doi:10.3389/frcha.2026.1686491)
Supplement: Additional File 5 — Standardized interview protocol for the midpoint-visit (PDF 271 kb). [file Datasheet5.pdf]

(5) Leitfaden und Dokumentation zum Abschlussgespräch

**Verantwortliche**:<br>(Prüfer:in/ Study Nurse)	Wählen Sie ein Element aus.	Fragen per Mail an:<br>studie.somnio.kiel@uksh.de

**Screening Nr.**: SOMJU-K01-	**Datum**:

Ziel des Leitfadens

Der Leitfaden zielt darauf ab, dass die telefonischen Interviews mit den Studienteilnehmenden standardisiert und einheitlich ablaufen. So wird sichergestellt, dass alle Studienteilnehmenden gleichbehandelt werden.

Wichtige Hinweise für das Interview

- Es ist wichtig, die Vorgaben des Leitfadens zu befolgen und am Ende des Gesprächs die Befolgung des Leitfadens schriftlich mit Unterschrift zu bestätigen.
- Sollten die Teilnehmenden medizinische/therapeutische Fragen zur Behandlung stellen, sollen die Teilnehmenden darauf hingewiesen werden, dass
  - im Rahmen des Gesprächs keine Therapie/Behandlung stattfinden kann.
  - im Rahmen des Gesprächs vorrangig organisatorische Fragen zur Studie oder allgemeine Verständnisfragen beantwortet werden können.
  - das Gespräch der Erfassung wichtiger Informationen für die Studie dient.
  - sich die Teilnehmenden mit medizinischen Fragen bitte an die Studienärztin oder ihre/n Behandler/Behandlerin wenden sollen.
- Sollten Teilnehmende aus der Interventionsgruppe technische Fragen oder Verständnisfragen zum Produkt haben, können diese, soweit möglich, beantwortet werden. Falls eine Beantwortung nicht möglich ist, können die Teilnehmenden an den mementor Support verwiesen werden.
- Sollten Teilnehmende aus der Kontrollgruppe Verständnisfragen zu ihrer Intervention haben, können diese, sofern möglich, ebenfalls beantwortet werden.
- Es ist bei Rückfragen zur Intervention darauf zu achten, dass die Teilnehmenden nicht beeinflusst (z.B. Motivation zur Nutzung ihrer Intervention) werden

Einleitung

Herzlich willkommen! Das Abschlussgespräch wird ungefähr ___ Minuten dauern (IG: 60, KG:20)

- Ziel des Gesprächs ist es, Informationen für die Studie zu sammeln.
- Im Rahmen des Gesprächs wird keine Behandlung / Therapie (Wenn konkrete medizinische Fragen vorliegen, sollten diese mit dem/der Behandler:in besprochen werden.)

Ablauf: Ich werde Dir jetzt zunächst einen kurzen Fragebogen freigeben. Danach ein paar Fragen zu dem Befinden in den letzten 6 Wochen stellen.

Dokumentation Erhebung t2

Wir schicken dir jetzt wieder den Link zu deinen Fragebögen zu.

Wichtiger Hinweis: Es dürfen den Teilnehmenden keine Vorgaben, Hinweise oder Anmerkungen zur Beantwortung der Fragen gegeben werden. Während des Ausfüllens darf nicht mit den Teilnehmenden gesprochen werden. Lediglich bei Unklarheiten oder Verständnisfragen seitens der Teilnehmenden darf während des Ausfüllens gesprochen werden.

- Die Teilnehmenden sollen über den Ablauf aufgeklärt werden (Fragebogen per Mail zugeschickt, kurz Bescheid geben sobald Fragebogen angekommen ist) und folgende Hinweise erhalten:
  - Beim Ausfüllen des Fragebogens sollen sich Teilnehmende ganz auf eigene Eindrücke und Erfahrungen stützen. Sollen sich in Ruhe Zeit dafür nehmen.
  - Es gibt keine richtigen oder falschen Antworten.
  - Es ist wichtig, dass der Fragebogen unabhängig und ungestört sowie ohne Beeinflussung von Interviewer:in oder anderen Personen ausgefüllt wird.
  - Während des Ausfüllens wird der/die Interviewer:in nichts proaktiv sagen, aber weiterhin im Online-Meeting bleiben, sollten Rückfragen aufkommen.
- Sobald die Mail mit dem Fragebogen bei den Teilnehmenden angekommen ist, soll der Fragebogen geöffnet werden und der/die Teilnehmende beginnt eigenständig das Ausfüllen der Fragen, sobald er/sie bereit ist, während der/die Interviewer:in nur bei Rückfragen aktiv wird.

Wenn der Fragebogen vollständig ausgefüllt ist, wird die Nachfrage gestellt, ob der Fragebogen vollständig ausgefüllt und abgeschickt wurde. Falls ja, geht es anschließend weiter mit der Abfrage des Befindens in den letzten 6 Wochen.

☐ Zusendung der Fragebögen für t2 an Teilnehmer:in	Datum:
☐ Fragebögen durch Teilnehmer:in ausgefüllt	Datum:

Anmerkung:
Die Daten aus den Fragebögen der t2-Erhebung liegen nach dem Ausfüllen durch die Teilnehmenden direkt im eCRF vor.

Fragen zu AEs und Veränderungen der Umstände

**Fragen zu deinem Befinden in den letzten sechs Wochen (seit dem Zwischengespräch)**		
Hattest Du in den letzten 6 Wochen Schulferien?	☐ Nein<br>☐ Ja (Start: Ende:)	
**Frage nach unerwünschten Ereignissen***	Ja	Nein
Sind Dir während der letzten sechs Wochen (seit dem Zwischengespräch) ungewöhnliche Dinge passiert?	☐	☐
Warst Du in den letzten sechs Wochen (seit dem Zwischengespräch) krank oder hast Du Dich krank gefühlt?	☐	☐
Falls Ja: Welche Symptome hattest Du? ☐ Kopfschmerzen ☐ Schwindel ☐ Erkältung ☐ Vermehrte Müdigkeit ☐ Andere: ____________________		
Kam es in den letzten sechs Wochen (seit dem Zwischengespräch) zu Unfällen (z.B. im Straßenverkehr, Zuhause oder in der Schule)	☐	☐
Hast Du Dich in den letzten sechs Wochen (seit dem Zwischengespräch) vermehrt traurig, ängstlich oder erschöpft gefühlt?	☐	☐
Fragen, um psychische Symptome wie z.B. vermehrte Gereiztheit, Konzentrationsstörungen o.ä. zu erfassen:		
1. Haben sich Deine schulischen Leistungen in den letzten sechs Wochen (seit dem Zwischengespräch) verschlechtert?	☐	☐
2. Hast Du dich in den letzten sechs Wochen (seit dem Zwischengespräch) vermehrt mit Deinen Sorgeberechtigten oder Freund:innen gestritten?	☐	☐
Falls Ja bei 1. Oder 2.: Sind diese auf Veränderungen des psychischen Zustandes der Teilnehmenden zurückzuführen?<br>Wenn ja, welche:	☐	☐

* falls es zu einem unerwünschten Ereignis gekommen ist: AE-Bogen ausfüllen

**Beschreibung**<br>(Betroffene Person, Start-/Stoppdatum, Intensität, Schwerwiegend (SAE?), Zusammenhang mit dem Prüfprodukt, Ergriffene Maßnahme, Konsequenz)

Änderungen Begleitmedikation/-therapie					Ja	Nein
Gibt es Änderungen in der Begleitmedikation?					☐	☐
Falls ja:						
Name Med.	Indikation	Dosis [Einheit]	Einnahme-frequenz	Darreichungs-form & Weg der Einnahme	Startdatum	Stoppdatum
						☐ Andauernd
						☐ Andauernd
Anmerkung:						

			Ja	Nein
Gibt es Änderungen in den begleitenden Therapien?			☐	☐
Falls ja:				
Leitdiagnose:	Insomnie schon Thema?	Setting:	Startdatum:	Stoppdatum:
	☐ Ja<br>☐Nein	Wählen Sie ein Element aus.		☐ Andauernd
Anmerkung:				

Maßnahmen zur Verbesserung des Schlafes

	Ja	Nein
Gibt es Änderungen in sonstigen Maßnahmen zur Verbesserung des Schlafs? (z.B. Alkohol, Cannabis, psychologische oder pharmakologische Maßnahmen, die nicht unter Begleitmedikation oder begleitende Therapie fällt)<br>Falls ja, welche:	☐	☐

Abfrage nach Device Deficiencies

Frage nach Device Deficiencies	Ja	Nein
Sind Produktmängel (App bzw. Flyer) aufgetreten?	☐	☐

Ärztliches/therapeutisches Gespräch zum medizinischen Bericht (nur IG)

☐ Entfällt (VP in der Kontrollgruppe)

**Hinweis**
Mit den Teilnehmenden der **Interventionsgruppe** findet im Rahmen der Abschlussvisite das Abschlussgespräch/die Verlaufskontrolle unter Verwendung des medizinischen Berichts aus der somnio junior App statt. Hierzu generiert der/die Teilnehmende in der App den medizinischen Bericht, teilt seinen Bildschirm oder sendet das PDF per Mail an das Studienzentrum. So kann der Bericht im Rahmen der Abschlussvisite besprochen werden.<br><br><u>Folgende ärztliche/therapeutische Tätigkeit findet im Rahmen des Gesprächs statt:</u><br>Auswertung und Verlaufskontrolle: Überprüfung des medizinischen Berichts über die Entwicklung behandlungsrelevanter Parameter (z.B. Schlafparameter), und Fortschritt des Programms sowie Bestimmung des therapeutischen Erfolgs und Festlegung weiterer Maßnahmen.
**Teilleistungen der ärztlichen/therapeutischen Tätigkeit**
• Auswertung und Interpretation der relevanten Schlafparameter (z.B. Schlafscore, Schlafeffizienz, Schlafqualität, Schlafdauer, Wachzeiten) zur Bewertung der Entwicklung der Insomnie und ihrer Symptome in Verbindung mit dem Fortschritt in der App<br>• Identifikation von Auffälligkeiten/Besonderheiten bei der Entwicklung der Insomnie und ihrer Symptome<br>• Bewertung der Auswirkungen der Entwicklungen (z.B. auf therapeutische, familiäre, soziale und schulische/berufliche Aspekte)<br>• Bewertung des Therapiefortschritts/-erfolgs<br>• Identifikation von weiteren möglichen medizinischen Maßnahmen für die weitere Therapie, z.B. Fortsetzung der (digitalen) KVT-I<br>• Besprechen der o.g. Punkte mit dem/der Patient:in und Festlegung der weiteren Behandlung gemeinsam mit dem/der Patient:in im Sinne des Shared Decision Makings<br>• Beantwortung von Fragen des/der Patient:in zur Therapie mit der App und der weiteren Behandlung
**Folgende Bestandteile des Gesprächs wurden vorgenommen**
☐ Die oben beschriebene ärztliche/therapeutische Tätigkeit mit den relevanten Teilleistungen wurde erbracht.
Das Gespräch hat _________ Minuten in Anspruch genommen.

Semistrukturiertes Interview zur Einstellung zum Prüfprodukt (IG)

☐ Entfällt (VP in der Kontrollgruppe)

**Semistrukturiertes Interview** zur Einstellung zum Prüfprodukt (Interventionsgruppe)			
"In den letzten 12 Wochen hattest Du die Möglichkeit, an der digitalen Intervention "somnio junior" teilzunehmen. Gerne möchte ich Dich nun zu Deinen Erfahrungen mit "somnio junior" befragen. Wenn Du eine Frage nicht beantworten möchtest, können wir diese gerne überspringen."			
Frage	Notizen		
Wie war Deine Erfahrung mit der App als digitales Schlaftraining?			
Findest du das Programm verständlich?	☐ Ja	☐ teilweise	☐ nein
Findest Du die Sprache verständlich?	☐ Ja	☐ teilweise	☐ nein
Erkennst Du Dich in den Situationen, die beschrieben wurden, wieder?	☐ Ja	☐ teilweise	☐ nein
Hattest Du i.d.R. genug Zeit, die App zu nutzen?	☐ Ja	☐ teilweise	☐ nein
War die Menge an Informationen im Programm angemessen?	☐ zu viel	☐ genau richtig	☐ zu wenig

**Anwendungsform**	
Wie hast Du die Anwendung genutzt?	☐ gar nicht<br>☐ zwischendurch (in Bus/Bahn/Wartezeiten)<br>☐ an einen festen Zeitpunkt (extra Zeit genommen)<br>☐ teils-teils<br>☐ Sonstiges:
Auf welchem Endgerät hast Du die Anwendung genutzt	☐ an einem Smartphone<br>☐ an einem Tablet<br>☐ überwiegend an einem Smartphone<br>☐ teils-teils<br>☐ überwiegend Browser-basiert<br>☐ Browser-basiert<br>☐ Sonstiges:
Anmerkungen:	

Olivia bzw. Albert sollte Dich als digitaler Schlafexperte/digitale Schlafexpertin durch das Programm leiten. Hast Du die Arbeit mit Deinem digitalen Coach als hilfreich erlebt?	☐ Ja	☐ teilweise	☐ nein
Was hat Dir an den Modulen im Training besonders gefallen? Warum?			
Was hat Dir besonders geholfen?			
Gibt es etwas, was Dir nicht gefallen hat? Warum?			

Hat sich Deiner Meinung nach Dein Schlaf verbessert oder verschlechtert?	
Was glaubst Du, womit könnte diese Veränderung zusammenhängen?	
Spielt „Angst vor dem Schlafengehen“ bei Dir eine Rolle und hat sich das im Verlauf der Studie verändert?	
An welchen Stellen ist es Dir schwer gefallen, die Therapie, wie besprochen, umzusetzen?<br><br>Anmerkung:	☐ Modul 1 (Einführung)<br>☐ Modul 2 (Das Schlaftagebuch)<br>☐ Modul 3 (Schlafwissen)<br>☐ Modul 4 (Kreislauf der Insomnie)<br>☐ Modul 5 (Schlafzeiten)<br>☐ Modul 6 (Schlafverhalten 1)<br>☐ Modul 7 (Entspannung)<br>☐ Modul 8 (Schlafverhalten 2)<br>☐ Modul 9 (Achtsamkeit)<br>☐ Modul 10 (Gedanken)<br>☐ Modul 11 (Alltagsentscheidungen)<br>☐ Modul 12 (Rückblick)

Wenn Du eine Schlaf-App für Jugendliche selber entwickeln könntest, was würdest Du anders oder besser machen?			
Welche Informationen hättest Du gerne zusätzlich gehabt? Was fehlt Dir an Tipps und Inhalten?			
Was war für dich unnötig bzw. zu viel?			
Würdest Du in Zukunft eine digitale oder eine persönliche Therapie bevorzugen?	☐ Digital	☐ Persönl.	☐ weiß nicht
Warum?			
Würdest du die App einem Freund oder einer Freundin weiterempfehlen?	☐ Ja	☐ Nein	☐ weiß nicht
Gibt es noch etwas, das Du gerne mit uns besprechen möchtest?<br>Hast Du noch Fragen an uns?			

**Anmerkungen**
Empfohlene Nachsorge nach Abschluss der klinischen Prüfung (siehe Prüfplan):<br>• Es wird empfohlen, den medizinischen Bericht dem Hausarzt zur Verfügung stellen.<br>• Auf Wunsch: Brief zur Bestätigung der Teilnahme an der Studie<br>Teilnehmer/-in wünscht Bestätigung ☐ Ja ☐ Nein

Abschluss des Gesprächs

**Gutscheinausgabe**
☐ Gutschein verschickt (per Mail)<br>☐ Teilnehmer Gutschein erhalten

**KG: Somnio junior Zugang**
☐ TN hat Zugangscode erhalten<br>☐ entfällt (TN in IG)

**Verabschiedung**
Vielen Dank für Deine/Eure Zeit. Wir sind jetzt fertig.

**Anmerkungen**

**Angabe zum Befolgen des Leitfadens**
☐ Die Vorgaben des Leitfadens wurden befolgt<br>☐ Die Vorgaben des Leitfadens wurden mit Abweichungen befolgt<br>Angabe der Abweichung:<br>Begründung:

Unterschrift: X ______________________

Prüfer:in/ Study Nurse
