# Supplementary material for: Digital treatment for insomnia in adolescents: study protocol for a randomized controlled trial comparing digital cognitive behavioral therapy for insomnia to sleep hygiene
Source: Front Child Adolesc Psychiatry. 2026 May 1;5:1686491. doi: 10.3389/frcha.2026.1686491 (PMC13176156; doi:10.3389/frcha.2026.1686491)
Supplement: Additional File 6 — Standardized interview protocol for the post-visit (PDF 326 kb). [file Datasheet6.pdf]

SPIRIT 2013 Checklist:
Recommended items to address in a clinical trial protocol and related documents*

Section/item	Item No	Description
**Administrative information**		
Title	1	„Somnio junior“ – study protocol of a randomized controlled clinical trial for evaluation of a digital cognitive behavioural therapy aiming to reduce symptoms of insomnia in adolescents after three months of use.
Trial registration	2a	German Clinical Trials Register<br>Identifier: DRKS00033527
	2b	Not applicable, registered at DRKS
Protocol version	3	• Issue date: 20.05.2025<br>• Protocol Version 2.3<br>Revision chronology:<br>• 06.01.2025: Initiation with protocol version 2.2
Funding	4	The digital application somnio junior is a product of the company mementor DE GmbH. This company also funds the clinical trial.The sponsor was involved in the design and conduct of the study but did not have contact with participants at any point. The sponsor was not involved in data collection.
Roles and responsibilities	5a	Beke Ralfs (Investigator)[1], Sarah Fee Meschkat (Investigator)[1], Telke Schoone (Physician)[1,2], Hannah Brauer (Counseling)[1], Jan-Henrik Rieck (Physician)[2], Manuel Munz (Physician)[2], Leonie Maurer (Trial statistician and conceptualisiation)[3], Charlotte Müller (Organisational management)[3], Alexander Prehn-Kristensen (Supervising Investigator)[1,3]<br>1. Institute of Child and Adolescent Psychiatry, Centre for Integrative Psychiatry, School of Medicine, Christian-Albrechts University Kiel, D-24105 Kiel, Germany.<br>2. Clinic for Child and Adolescent Psychiatry, Psychotherapy and Psychosomatics, Centre for Integrative Psychiatry, School of Medicine, D-24105 Kiel, Germany<br>3. Mementor DE GmbH, D-04229, Leipzig, Germany<br>4. Department of Psychology, Faculty of Human Sciences, MSH Medical School Hamburg - University of Applied Sciences and Medical University, D-20457 Hamburg, Germany

	5b	mementor DE GmbH, Karl-Heine-Straße 15, 04229 Leipzig,<br>Phone: +49 341 581 444 33<br>E-Mail: info@mementor.de
	5c	The sponsor was involved in study design and will be involved in data analysis and interpretation and writing of the report, with the analysis being the only part where the sponsor will have ultimate authority. Data collection and management is solely performed by the investigators and data manager, respectively. Decision to submit the report for publication does not lie within sponsor responsibility.
	5d	The study centre is located in the Institute of Child and Adolescent Psychiatry, Centre for Integrative Psychiatry, School of Medicine, Christian-Albrechts University Kiel. The investigators are responsible for data collection and study organisation. Data management is performed by the Centre of Clinical Trial in Kiel.
**Introduction**		
Background and rationale	6a	Insomnia is highly prevalent among adolescents and is associated with various mental and physical disorders and problems of functioning in different areas. Cognitive behavioural therapy is well supported by evidence and the recommended method of treatment, but too few adolescents receive access to this guideline-based treatment. Tackling this care gap, digital applications implementing contents of cognitive behavioural therapy show promising results and could be a more economic, low-threshold form of treatment.
	6b	The current study compares the use of the digital application *somnio junior* (digital CBT-I) with general information on sleep hygiene, which participants of the CG will receive as a digital flyer.
Objectives	7	It is hypothesized that the insomnia severity (primary endpoint) will decrease in participants of the IG compared to the CG. Further, a decline in symptoms of depression, anxiety, daytime sleepiness and an increase in health-related quality of life (secondary endpoints) is expected in the IG compared to the CG.
Trial design	8	The current study is a monocentric, national, prospective randomized controlled trial. A central stratified block randomization with variable block size randomized participants after baseline to IG and CG. Participants are equally distributed between IG and CG with regard to gender.

Methods: Participants, interventions, and outcomes

Study setting	9	The study center is the Institute of Child and Adolescent Psychiatry at the Center for Integrative Psychiatry, School of Medicine in Kiel, Germany. The clinical trial is conducted entirely in an online setting, thus adolescents from all over Germany can participate. All visits and interviews are held via the video conferencing platform BigBlueButton (BBB) hosted by the University´s Computing Center and questionnaires are filled out online directly via the Electronic-Data-Capture-System (EDC). Information material about the study is openly available on the products website, there are advertisements on social media and a list of psychiatric institutions, clinics and practitioners exhibit flyers in their facilities and inform patients struggling with insomnia about the possibility of participating in the clinical trial.
Eligibility criteria	10	<u>Inclusion criteria</u><br>- Age between 14 and 17 years<br>- Informed consent in written form of participant and all legal guardians via DocuSign<br>- F51.0/G47.0 diagnosis OR DSM-V criteria for insomnia<br>- Access to either smartphone or tablet compatible with the application *somnio junior* and connection to the internet<br>- The participants are able to fully participate in the clinical trial and meet the requirements for participation<br>- Ability to speak and read the German language<br><br><u>Exclusion criteria</u><br>- Acute suicidal behaviour (self-disclosure)<br>- Epilepsy (self-disclosure)<br>- Bipolar disorder (self-disclosure)<br>- Addiction, including media-related disorders (self-disclosure)<br>- Acute psychiatric hospitalization in the past four weeks (self-disclosure)<br>- Organically caused sleep disorders, e.g. as a result of increased itching (in acute or chronic skin diseases such as neurodermatitis, lice infestation, scabies), pain or respiratory diseases (self-disclosure)<br>- Participation in another clinical study (self-disclosure)

Interventions	11a	The digital CBT-I intervention *somnio junior* is compared to advice on sleep hygiene. In both groups the treatment as usual (TAU) is continued.

Somnio junior

The digital application can be installed on a smartphone or tablet. Within the app, a digital sleep expert will guide users through different modules on different topics targeting their sleep problems, such as psychoeducational contents (sleep knowledge, information on sleep regulation, cycle of insomnia, stimulus control, sleep environment, daily activities, substance use, nutrition, dysfunctional thoughts on sleep-related issues, influence on everyday decisions on sleep), practical exercises (applying acquired knowledge on practical examples, relaxation, mindfulness) and bedtime restriction. Users are asked to fill out a sleep diary with a morning and evening protocol to monitor effects. The intervention (e.g. bedtime restriction) is adapted according to this data.

Advice on sleep hygiene ("Your top 10 rules for better sleep")

1. Behaviour in bed: Use the bed only for sleeping
2. 15-minute rule: If you can´t sleep after 15-20 minutes after going to bed, get up and do something quite until you feel sleepy
3. Bed & Lighting: Find a comfortable bed, be sure it´s dark in your bedroom
4. Temperature: Room temperature should not be too high or too low (around 17°C)
5. Noise during bedtime: Should be eliminated if possible
6. Betime Routines: May help with calming down in the evening
7. Mobile phone: Try reducing screentime to max. two hours daily
8. Nutrition: Avoid eating large and heavy meals late in the evening
9. Exercise: Helps with sleeping, try exercising 60 minutes a day
10. Substances: Avoid alcohol, cannabis or nicotine as sleep medication, since they harm sleep quality on the long term.

11b There are no criteria for modifying the intervention. However, as part of the regular intervention within the digital application, data on individual goals and data from the diary is used to adapt the programme to fit the individual needs.
If there is any indication that the safety of participants might be compromised, the intervention is discontinued.

11c To improve adherence, there will be a midpoint visit. Adolescents and parents are informed at baseline visit, that they will be contacted via telephone to find a date for midpoint and post assessment. Further, they are reminded via email a few days before visits.

	11d	Participants should continue any medical or therapeutic care, as they have before. All medication and therapy (and all changes) are documented.
Outcomes	12	The insomnia severity index (ISI) is used as the primary endpoint, because it was shown to adequately quantifying changes during insomnia therapy. It is measured at baseline, midpoint and post visit. Since the influence of insomnia and associated psychiatric disorders (f.e. depression and anxiety) is assumed to be bidirectional, changes in insomnia severity might also be associated with changes in depressive symptoms and anxiety, measured by the Patient Health Questionnaire-9 (PHQ-9) and the Generalized Anxiety Disorder (GAD-7), respectively. Further, daytime sleepiness is assumed to mediate between insomnia symptoms and daytime functioning. Effects of the intervention on daytime sleepiness is measured by the Stanford Sleepiness Scale (SSS). Lastly, the health-related quality of life is measured by the KINDL (Fragebogen zur Erfassung der gesundheitsbezogenen Lebensqualität bei Kindern und Jugendlichen). All of the related measures are measured at baseline and post visit.
Participant timeline	13	For time schedule of enrolment, interventions, assessments, and visits for participants see the following table.

	Study period				
				Post-allocation	
Time point	Enrollment	t0	Allocation	t1[1]	t2[2]
**Enrollment**					
Informed consent	X				
Eligibility screen	X				
Allocation			X		
**Intervention**					
Somnio junior + TAU			◆	—	◆
Sleep hygiene + TAU			◆	—	◆
**Assessments**					
Demographics		X			
Primary outcome (ISI)		X		X	X
Secondary outcomes		X			X
Diagnostic measures		X			
Medical history		X			
Medication tracking		X		X	X

Abbreviations: TAU Treatment as Usual; *ISI* Insomnia Severity Index
[1] t1: 6 weeks after baseline
[2] t2: 12 weeks after baseline

Sample size	14	Assuming that this outcome follows a normally distributed population and that linear mixed models rely on pairwise comparisons, the hypothesis will be tested using a two-sided t-test for independent samples within a parallel-group design. To detect a statistically significant group difference with a Standardized Mean Difference (SMD) of 1.07 (the lower confidence interval of the within-group effect size reported in a prior study on the efficacy of digital CBT-I in adolescents using the Insomnia Severity Index) with a power of 95% and an alpha level of 0.05, 24 participants per group are required (calculated using G*Power). Considering an anticipated dropout rate of 27%, a total of 66 adolescents should be randomized for the study.
Recruitment	15	Information material about the study is openly available on the products website, there are advertisements on social media and a list of psychiatric institutions, clinics and practitioners exhibit flyers in their facilities and inform patients struggling with insomnia about the possibility of participating in the clinical trial.

Methods: Assignment of interventions (for controlled trials)

Allocation:

Sequence generation	16a	A central stratified block randomization with variable block size (2-4) is selected as the randomization procedure. This way, participants are equally distributed between IG and CG regarding gender. Randomization will be implemented through the EDC by the Center of Clinical Trials (Zentrum für Klinische Studien; ZKS) in Kiel and its sequence is concealed until interventions are assigned.
Allocation concealment mechanism	16b	The allocation sequence is implemented in the background of the Electronic-Data-Capture-System (EDC). After filling out the demographic information (since participants are equally distributed between IG and CG with regard to gender), the system generates the allocation directly. To conceal the sequence, a block-design is used.
Implementatio n	16c	The allocation sequence is generated by the Data Manager. Participants are enrolled by the Investigators. The assignment to interventions is generated automatically based on the allocation sequence and demographic information (gender) by the EDC.
Blinding (masking)	17a	Due to the intervention characteristics, investigators and participants are unblinded. Data analysists are blinded.
	17b	Not applicable

Methods: Data collection, management, and analysis

Data collection methods	18a	Data on primary and secondary endpoints are collected directly via the EDC. Other data (demographic data, adverse events, …) are collected by the investigators using standardized protocols. All investigators are trained with these protocols to minimize deviations. The following questionnaires are used:<br><u>Primary endpoint:</u><br>• Insomnia Severity Index (ISI) was shown to adequately quantifying changes during insomnia therapy.<br>Metrics (German Version): Chronbach's alpha = 0.83; high construct validity: correlation to Pittsburgh Sleep Quality Index (PSQI) total score r = 0.79<br><u>Secondary endpoints:</u><br>• Patient Health Questionnaire-9 (PHQ-9) measures symptoms of the major depressive disorder.<br>Metrics: Cronbach alphas = 0.86 to 0.89; high Criteria validity by comparing results to structured interviews by mental health professionals<br>• Generalized Anxiety Disorder (GAD-7)<br>Metrics: High internal consistency (Cronbach alphas = 0.93), good construct validity (moderate correlations to depressive symptoms)<br>• Stanford Sleepiness Scale (SSS)<br>• Health-related quality of life in children and adolescents (KINDL)<br>Metrics: Cronbach's alpha = 0,85; good validity
	18b	To ensure participant retention and complete to follow-up, the participants and custodians are informed at baseline, when they will be contacted via phone to find a date for midpoint and post assessments. The investigation team uses a shared study calendar to ensure all visits are planned in time.
Data management	19	The data entry in the EDC is done by the Investigators. The source documents are stored digitally so that a re-check can be done. To promote data quality, automatic range checks are performed (if the item allows). Investigators have to safe changes and are then asked to doublecheck and sign the entry. Data is further checked for errors in transmission from source date in monitoring visits and on plausibility by the data manager.

Statistical methods	20a	Primary outcome:<br>A mixed effect linear model based on an unstructured covariance matrix will be fitted to the primary outcome data (ISI at 12 weeks), utilising 6- and 12-week timepoints. Participant will be included as random effects. Fixed effects will include randomized group, baseline ISI score, group, time and a time by randomized group interaction term to allow estimation of treatment effect at each timepoint.<br><br>Secondary outcome:<br>Continuous secondary outcomes with only two time point measures (baseline and 12-weeks) will be analyzed with ANCOVA models, adjusting for baseline variable. The estimated difference between the groups after 12 weeks will be derived using a linear contrast statement from the ANCOVA model.
	20b	Subgroup analyses will be conducted based on gender (female, male). Secondary count outcomes (e.g., number of responders, number of side effects, number of measures to improve sleep) will be analyzed using Chi-Square tests. For the primary outcome, insomnia severity, a responder analysis will be conducted based on the Minimal Clinically Important Difference (MCID) for the Insomnia Severity Index (ISI), defined as a change in ISI ≥ 8. This analysis will examine differences between groups. Similarly, responder analyses will be conducted for depression (change in PHQ-9 ≥ 5; Löwe et al., 2004) and anxiety measures (change in GAD-7 ≥ 3; Kroenke et al., 2019).
	20c	Missing data will be reported, including any available reasons for the missingness, and the patterns of missing data will be examined. The mixed-effects model inherently accounts for data assumed to be missing at random. Missing data, assuming confirmation of the "Missing at Random" (MAR) condition, will be estimated using multiple imputation.
**Methods: Monitoring**		
Data monitoring	21a	Monitoring will be performed by the Center of Clinical Studies (Zentrum für Klinische Studien, ZKS). During the intervention period of the clinical trial, a monitor will review the study at least three times. Further visits will be added, if any deviations occur. The monitor will report to the sponsor about any deviations. The monitor is independent from the sponsor with no competing interests.
	21b	After n=33 participants have completed the study, there will be interim analyses. However, there is no stop-criterion connected to this analysis. The trial will only be terminated if any major safety concerns arise.

Harms	22	Participants and custodians are informed that they should reach out to the investigators if any adverse events occur. Further, in the midpoint and post visit, participants are asked in detail about any adverse events that might have occurred since the last visit and about problems with the intervention. All adverse events are discussed and evaluated with the trial physician.
Auditing	23	The sponsor reserves the right to audit the investigative site.
**Ethics and dissemination**		
Research ethics approval	24	The study has been approved by the ethics committee of the medical faculty of the Christian-Albrechts-University in Kiel (16.07.2024). The study will be conducted in line with institutional requirements and local legislation.
Protocol amendments	25	Protocol amendments are communicated by the project manager (employee of the Center of Clinical Trials; Zentrum für Klinische Studien; ZKS) to the ethics committee of the medical faculty of the Christian-Albrechts-University in Kiel.
Consent or assent	26a	Informed consent will be obtained by the investigators via DocuSign after participants and custodians received a detailed education on all relevant aspects of the clinical trial by a physician.
	26b	Participants and custodians can decide separately from participation, if they allow an audio-recording of the standardized interview on their experience with the application done after the post visit.
Confidentiality	27	Every adolescents that is interested in participating will receive a participant code at first contact. After that, all data is saved using this code. There are only two documents using names: The informed consent is separately saved and stored and the list assigning names and contact information to the participant codes is filed safely. No study data can be directed to a certain person without this list.
Declaration of interests	28	APK reported receiving grants from the DFG, Innovation Funds of the Federal Joint Committee, Interreg Germany-Denmark, and Mementor by ResMed. For the other investigators on the study site there are no competing interests.
Access to data	29	The investigative site has full access to the data. The sponsor receives anonymized and blinded data for analysis. The sponsor has the authority to decide whether the anonymized data may be shared and with whom.
Ancillary and post-trial care	30	Not applicable

Dissemination policy	31a	The sponsor welcomes the publication of scientifically and clinically meaningful clinical trials resulting from the study. Therefore, publication of the results in scientific journals and/or at medical congresses is planned. To ensure appropriate publication, registration of the clinical trial in the DRKS registry portal (German Clinical Trials Register) is intended prior to the start of the trial. All publications will comply with data protection requirements. Neither the sponsor nor any person commissioned by the sponsor has the right to prevent publication, unless there are patent or copyright-related reasons. The sponsor must receive a draft summary or manuscript for review, editorial approval, and release prior to submission or presentation.
	31b	Active participation in the study is a prerequisite for authorship. No intended use of professional writers
	31c	No plans for granting public access to the full protocol, participant-level dataset, and statistical code
**Appendices**		
Informed consent materials	32	• Informed consent form for the adolescents<br>• Informed consent form for the custodians
Biological specimens	33	Not applicable

*It is strongly recommended that this checklist be read in conjunction with the SPIRIT 2013 Explanation & Elaboration for important clarification on the items. Amendments to the protocol should be tracked and dated. The SPIRIT checklist is copyrighted by the SPIRIT Group under the Creative Commons "Attribution-NonCommercial-NoDerivs 3.0 Unported" license.
